# Supplementary material for: Elevated atmospheric CO2 decreases methylmercury production in freshwater lakes
Source: Nat Commun. 2025 Dec 26;17:1037. doi: 10.1038/s41467-025-67788-0 (PMC12848011; doi:10.1038/s41467-025-67788-0)
Supplement: Supplementary file 1 — Supplementary Information [file 41467_2025_67788_MOESM1_ESM.pdf]

Supplementary information

Elevated atmospheric CO_2 decreases methylmercury production in freshwater lakes

Pei Lei[a,b,c,#], Jin Zhang[a,b,#], Ri-Qing Yu[d], Maciej Bartosiewicz[e,f], Chengjun Li[g], R. Iestyn, Woolway[h], Martin Tsz-Ki Tsui[i], Tao Jiang[j], Bo Meng[k], Raymond W. M. Kwong[l], Yuming Guo[m], Huan He[a], Xinghui Xia[n], Hongqiang Ren[b], Huan Zhong[b,*]

a. School of Environment, Nanjing Normal University, Nanjing, China

b. State Key Laboratory of Water Pollution Control and Green Resource Recycling, Nanjing University, Nanjing 210023, China

c. State Key Laboratory of Lake and Watershed Science for Water Security, Nanjing Institute of Geography and Limnology, Chinese Academy of Sciences, Nanjing 210008, China

d. Department of Biology, Center for Environment, Biodiversity and Conservation, The University of Texas at Tyler, Tyler, TX, USA

e. Department of Environmental Sciences, University of Basel, Basel, Switzerland

f. Institute of Geophysics, Polish Academy of Sciences, Warsaw, Poland

g. Institute of Environmental Research at Greater Bay Area, Key Laboratory for Water Quality and Conservation of the Pearl River Delta, Ministry of Education, Guangzhou University, Guangzhou China

h. School of Ocean Sciences, Bangor University, Menai Bridge, Anglesey, United Kingdom

i. School of Life Sciences, Chinese University of Hong Kong, Shatin, New Territories, Hong Kong SAR, China

j. Interdisciplinary Research Centre for Agriculture Green Development in Yangtze River Basin, College of Resources and Environment, Southwest University, Chongqing, China

k. State Key Laboratory of Environmental Geochemistry, Institute of Geochemistry, Chinese Academy of Sciences, Guiyang, China

l. Department of Biology, York University, Toronto, Canada

m. Department of Epidemiology and Preventive Medicine, School of Public Health and Preventive Medicine, Monash University, Melbourne, Australia

n. School of Environment, Key Laboratory for Water and Sediment Science, Ministry of Education, Beijing Normal University, Beijing, China

[#] P.L. and J.Z. contributed equally to this work.

* Corresponding Author: Huan Zhong*, State Key Laboratory of Water Pollution Control and Green Resource Recycling, School of the Environment, Nanjing University, Nanjing, China, Email: zhonghuan@nju.edu.cn

Supplementary Text 1 Influence of other environmental factors on CO_2-impacted Hg methylation

Although significant reductions in MeHg formation were observed under elevated CO_2 based on our empirical evidence and modelling results, there are additional factors that can regulate the Hg methylation process and contribute to uncertainties in predicting CO_2-impacted Hg methylation in lakes. These factors should be considered and investigated in future studies to reduce the uncertainties when extrapolating our results to real-world scenarios.

First, we quantified the responses of microbial Hg methylation to elevated CO_2 levels over a two-month incubation under dark conditions. This design intentionally excluded photodegradation, which is strongly suppressed in eutrophic lakes due to the light-shading effect of AOM [1], but remains important in oligotrophic or clear-water systems. While MeHg concentrations would be lower under light exposure compared to dark incubation, the relative reduction rates are expected to remain similar under both ambient (420 ppm) and elevated (1000 ppm) CO_2, because DOC concentrations were not significantly altered by CO_2 elevation ($p > 0.05$, Supplementary Fig. T1-1). Thus, the observed inhibitory effect of elevated CO_2 on net MeHg production is robust even when photodegradation is considered. Future work should incorporate *in-situ* light regimes to extend applicability to a broader range of lake types. In addition, further research is needed to assess long-term responses, as Hg-

methylating microbes such as methanogens may gradually acclimate to elevated CO_2 and other environmental changes during prolonged incubation [2,3].

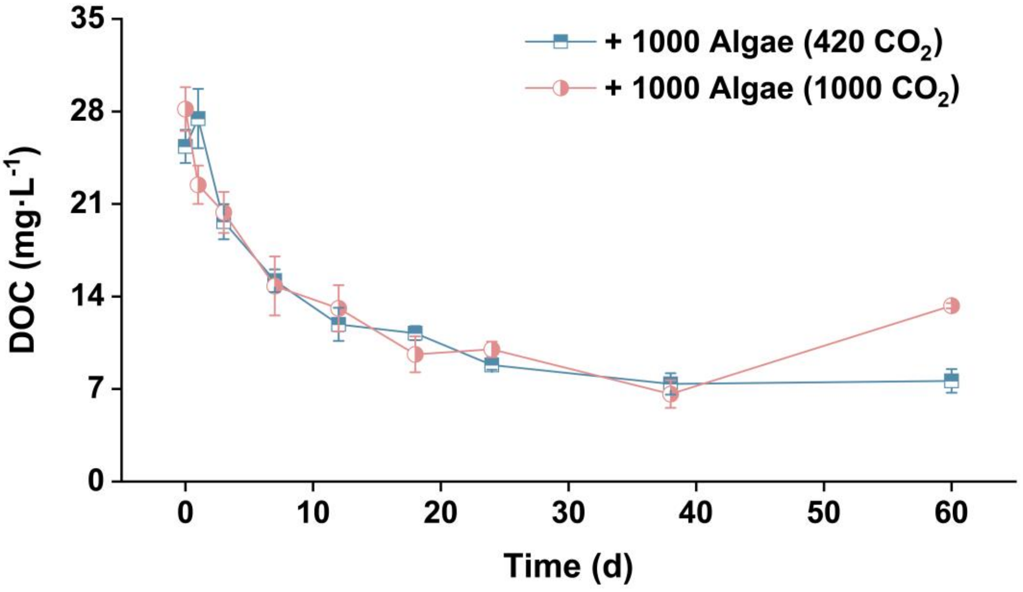

Supplementary Fig. T1-1 Dynamic changes of DOC levels (mean ± SD; *n* = 4) during incubation under ambient CO_2 (420 ppm) or elevated CO_2 (1000 ppm) levels.

Another key factor that may interfere with the CO_2-MeHg nexus in lake waters is sediments, which represent the largest pool of Hg in aquatic ecosystems. Predicting climate-driven changes in freshwater MeHg levels also requires understanding how elevated CO_2 affects sedimentary MeHg production and water-sediment MeHg fluxes. Our recent parallel study on coastal marine sediments (East China Sea) reveals that elevated CO_2 (1000 ppm) also suppresses MeHg production in sediments, with reductions ranging from 54% to 77% compared to ambient conditions (420 ppm) [4]. However, considering the partitioning of MeHg between sedimentary solid and water phases (e.g., with partition coefficient K_d of 10^3–10^5 L·kg^{-1} [5]), the CO_2-impacted

MeHg from sediment represents only a small fraction (e.g., <12%) of the total dissolved MeHg observed in lake water [6]. Nevertheless, this consistency across water column and sediment systems suggests that elevated CO_2-induced inhibition of microbial Hg methylation may represent a generalizable pattern across aquatic environments, reinforcing the broader relevance of our findings. Future studies will explore how elevated atmospheric CO_2 indirectly affects sedimentary methylation (e.g., via altered organic matter export from the water column or porewater chemistry) to better quantify total lake MeHg budgets under climate change.

Our model incorporates DOC as a parameter primarily due to the availability of global DOC datasets, which enable broad-scale extrapolation. However, DOC represents a heterogeneous pool encompassing both autochthonous and allochthonous fractions that may differentially regulate Hg methylation and demethylation [7]. Specifically, algae-derived AOM, particularly in eutrophic lakes, is a functionally distinct driver of microbial Hg methylation [8], while soil-derived SOM typically exerts weaker effects [7]. To clarify how AOM contributions modulate CO_2-driven inhibition of MeHg production, we conducted additional experiments with mixed DOM sources: pure AOM, pure SOM, and AOM:SOM ratios of 80:20, 50:50, and 20:80. Elevated CO_2 significantly suppressed net MeHg production only when AOM constituted $\geqslant$ 50% of the DOM pool ($p < 0.001$). The inhibition rate declined from 75% (pure

AOM) to 68% (80:20), 52% (50:50), and became negligible (<10%) when AOM was <50% (Supplementary Fig. 4). These findings demonstrate that while our model may overestimate MeHg declines in lakes dominated by terrestrial DOM, the deviation for eutrophic lakes (the principal focus of this study) is moderate. After normalizing by the AOM:SOM ratio, projected global inhibition ranges were refined from 31–86% (mean 50%) to 33–74% (mean 47%).

Furthermore, elevated atmospheric CO_2 typically co-occurs with global warming, which may alter microbial processes involved in Hg methylation and demethylation [9]. To clarify this interplay, we conducted supplementary experiments simulating different climate scenarios: "Control" (25°C, ambient 420 ppm CO_2), "Warming" (29.4°C, ΔT=4.4°C, consistent with SSP5-8.5 projections for 2100, ambient 420 ppm CO_2), "Elevated CO_2" (25°C, elevated CO_2 of 1000 ppm), and "Warming × Elevated CO_2" (29.4°C, elevated CO_2 of 1000 ppm). Results showed that warming alone increased net MeHg production by 17–37% ($p < 0.01$), likely due to stimulated microbial activity, while elevated CO_2, either alone or combined with warming, significantly suppressed MeHg production (26–74% reduction vs. Control) with comparable inhibition rates (33–76%, $p > 0.05$, Supplementary Fig. 5). These findings indicate that the inhibitory effect of elevated CO_2 on microbial Hg methylation outweighs the stimulatory effect of rising temperature, supporting the robustness of our model in projecting global MeHg trends despite concurrent warming.

Supplementary Text 2 Elevated CO_2 favors hydrogenotrophic methanogenesis via stimulated fermentative H_2 production

In the process of methanogenesis, H_2 plays a crucial role, and its availability is a key factor regulating the community structure of hydrogenotrophic and acetoclastic methanogens [10]. Hydrogenotrophic methanogenesis occurs through the reaction $CO_2 + 4\ H_2 \rightarrow CH_4 + 2\ H_2O$, producing approximately 1.5–2.5 ATP per mole of methane. In contrast, acetoclastic methanogenesis ($CH_3COOH \rightarrow CH_4 + CO_2$) yields only about 1 ATP per mole [11,12]. It is evident that hydrogenotrophic methanogenesis has a significant advantage in energy output and is more favored when substrates such as H_2 and CO_2 are abundant. However, the supply of H_2 is often limited because acetate fermentation typically produces only 2 moles of H_2 per mole, which becomes a bottleneck for the growth of hydrogenotrophic methanogens in natural systems [13].

In this study, the observed shift of the methanogen community towards hydrogenotrophic types (e.g., *Methanobacterium*) under high CO_2 conditions may be since high CO_2 stimulates fermentative bacteria such as *Clostridia* and *Enterobacter* to degrade algal organic matter. Through dark fermentation (e.g., glucose $\rightarrow$ 2 acetate + 2 CO_2 + 4 H_2), the substrate flux is adjusted to favor H_2 production over acetate, thereby alleviating H_2 limitation and enabling hydrogenotrophic methanogens to outcompete acetoclastic populations (e.g., *Methanosarcina*). Nevertheless, due to technical limitations in our culture

system, specifically the low solubility of H_2 in water and its rapid consumption by methanogens, it was difficult to capture stable concentrations in the closed microcosm system, resulting in the failure to successfully quantify headspace H_2. However, the consistent shift towards hydrogenotrophic methanogens across all treatments strongly indicates that H_2 availability was sufficient to support their growth under high CO_2 conditions. Given the critical role of H_2, future research should focus on the following aspects: using more sensitive detection methods to track H_2 dynamics in real-time to quantify its coupling with changes in the methanogen community; identifying key fermentative taxa responsible for H_2 production under high CO_2 conditions; and measuring ATP yields in pure cultures of hydrogenotrophic and acetoclastic methanogens to directly verify energy trade-offs, thereby further clarifying the role and mechanism of H_2 in methanogenesis and community structure regulation under climate change.

Supplementary Text 3 Iron reducing bacteria, sulfate reducing bacteria or acidification were not mainly responsible for the reduced MeHg formation under elevated CO_2

The other major Hg methylating microbes—iron reducing bacteria (FeRB) and sulfate reducing bacteria (SRB)—may have a minor impact on the observed reduction in MeHg production under elevated CO_2. This is evidenced by the lack of difference in the levels of dissolved sulfate (indicating sulfate reduction and hence the activities of SRB [14]) and dissolved Fe (indicating iron reduction and thus the activities of FeRB [15]) between "Ambient CO_2" and "Elevated CO_2" treatments ($p > 0.05$, Supplementary Fig. T3-1a), particularly during the initial 12-day incubation. At the same period, however, significant decreases in MeHg production (60% to 93%) under elevated CO_2 were observed. These results indicate that elevated CO_2 does not affect the activities of SRB and FeRB, suggesting that these two groups of microbes might play minor roles in the decreases in MeHg production during this period. However, after 18 days of algal decomposition, elevated CO_2 caused a significant iron reduction ($p<0.001$, Supplementary Fig. T3-1b), implying the enhanced activities of FeRB should not be the main reason for the inhibited MeHg production during this period. Throughout the incubation period, elevated CO_2 did not promote the reduction of sulfate, indicating that the activities of SRB were not affected by elevated CO_2 and thus changes in SRB activities may not be responsible for the

decreases in MeHg formation.

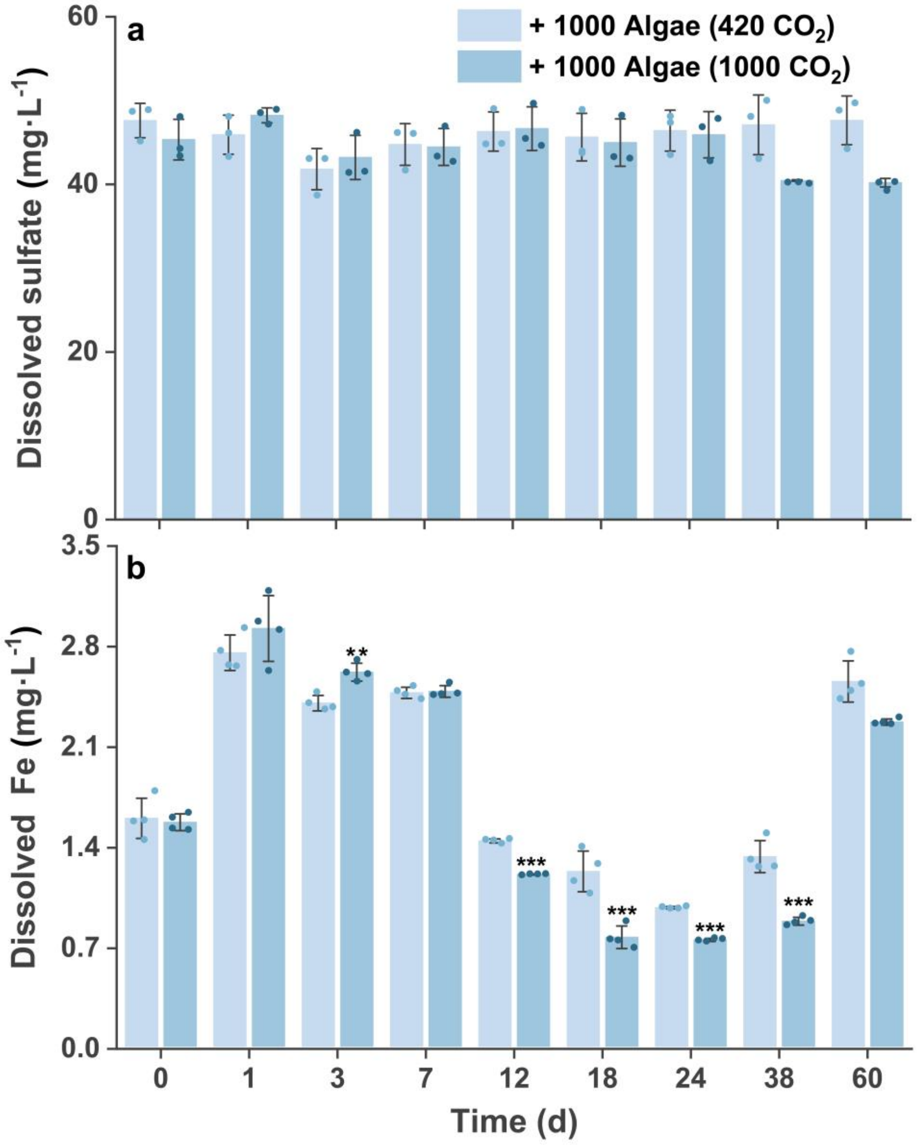

Supplementary Fig. T3-1 Dynamic changes in the concentrations of (a) dissolved sulfate (mean ± SD; *n* = 3) and (b) dissolved Fe (mean ± SD; *n* = 4) in water under ambient CO_2 (420 ppm) or elevated CO_2 (1000 ppm) conditions during a 60-day incubation. "+1000 Algae": unfiltered lake water (40 mL) amended with 0.005 g of algal biomass from Chaohu Lake.

Moreover, the decline in MeHg formation under elevated CO_2 may not be attributed to acidification and thus alterations in Hg bioavailability [16,17], a key

determinant of microbial Hg methylation in addition to microbes [18]. This is because during the initial stages of algal decomposition (prior to Day 7), elevated CO_2 did not cause significant changes in pH value (p>0.05, Supplementary Fig. T3-2). However, in the middle and later stages (after 7 days), the pH level of the solution under elevated CO_2 reduced significantly due to CO_2-induced water acidification (p<0.001, Supplementary Fig. T3-2), possibly leading to an increase in Hg bioavailability and facilitated Hg methylation [19]. Yet, despite this, elevated CO_2 was found to hinder the formation of MeHg during this period (p<0.001, Fig. 2c), suggesting that acidification, and thus possible increases in Hg bioavailability, may not be the key factor responsible for the reduction in MeHg formation.

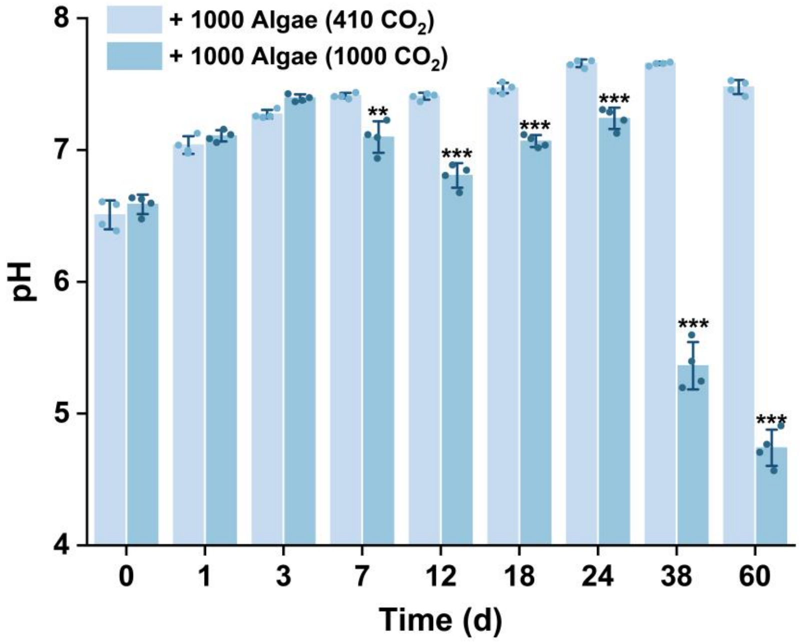

Supplementary Fig. T3-2 Dynamic changes in pH values (mean ± SD; n = 4) in water under ambient CO_2 (420 ppm) or elevated CO_2 (1000 ppm) conditions during a 60-day incubation.

Supplementary Text 4 Projected decreases in MeHg production in global lake water under elevated CO_2 based on observational database

To assess the potential influence of the predicted rise of CO_2 to 1000 ppm on MeHg levels in freshwater lakes worldwide, a model evaluation was undertaken by utilizing the relationships between CO_2-inhibited net MeHg production, as established in this study (described in the section of Methods), and existing global data on the observed ambient DOC and MeHg levels in freshwater lakes (summarized in Supplementary Table 3). While acknowledging certain uncertainties in our model estimation (uncertainties in our model estimation discussed below), our preliminary findings suggests that elevated CO_2 might result in reductions ranging from 34% to 79% (averagely 47%) in the MeHg levels in lakes worldwide, especially those with high DOC contents (Supplementary Fig. T4-1). In lakes characterized by high ambient DOC levels (averaging 23.4 $mg \cdot L^{-1}$) and MeHg contents (averaging 0.26 $ng \cdot L^{-1}$), which represent approximately 10% of the lake examined, MeHg levels are projected to decrease by 60% to 80% with elevated CO_2 levels. With the expected rise in CO_2, around one-third of global lakes are anticipated to undergo a reduction in MeHg levels ranging from 30% to 40%, another third from 40% to 50%, while the remaining third experience a decrease exceeding 50%.

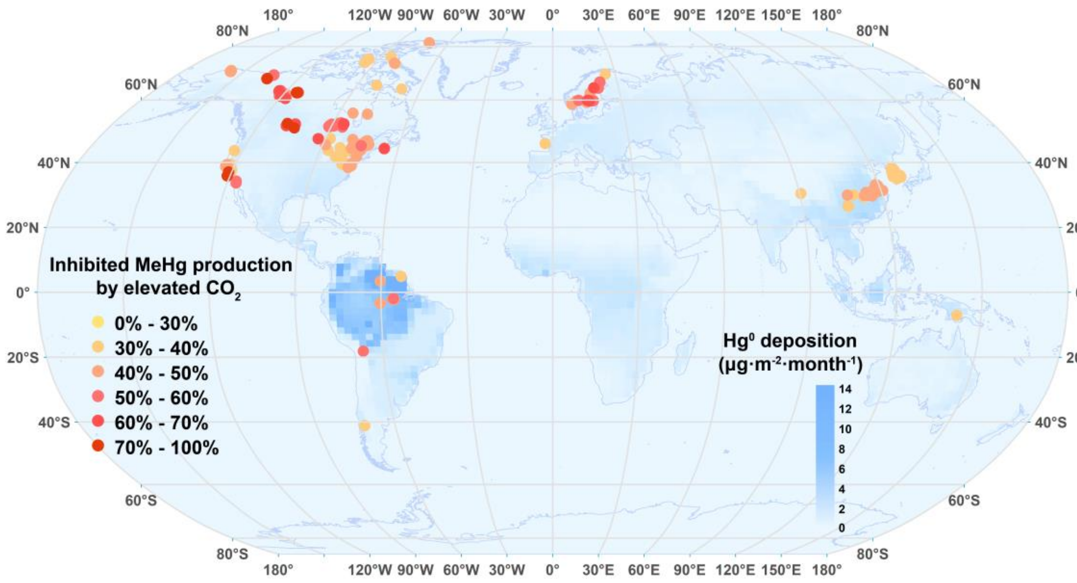

Supplementary Fig. T4-1 Projected decreases in MeHg production under elevated CO_2 conditions (1000 ppm in 2100) in global lakes based on observed ambient DOC and MeHg concentrations. A total of 277 samples were included in the analysis, comprising 229 samples of 49 publications from 2000 to 2023, which reported on MeHg and DOC concentrations in lake water columns, along with 48 samples collected from this study. The background DOC concentrations in 229 lake water samples did not exceed 30 $mg \cdot L^{-1}$, with an average of 9.51 $mg \cdot L^{-1}$. The initial dissolved MeHg concentrations in 229 lake water samples ranged from 0.001 to 2.9 $ng \cdot L^{-1}$, with an average of 0.14 $ng \cdot L^{-1}$. Information regarding global-scale deposition of Hg^0 was acquired from a recent report [20]. The global map was obtained from Natural Earth dataset (https://www.naturalearthdata.com/downloads/10m-physical-vectors/).

Monte Carlo simulation performed with Crystal Ball functions of Microsoft Excel (Microsoft Corporation, Microsoft Office Home and Student 2019) was used to quantitatively estimate the uncertainty of inhibited MeHg production.

Using the database obtained from previous published literature and observational data from this study, we generated 10,000 random values of MeHg levels for further prediction by employing the best fit lognormal distribution suggested by the Crystal Ball functions (Supplementary Fig. T4-2). Additionally, we generated 10,000 random values of DOC levels by assuming the lognormal distribution of the values in the actual lake environment (Supplementary Fig. T4-3). The models were run 10,000 times, following previous studies [8]. Median values and 90% confidence intervals (ranging from 5% to 95%) were generated to quantify uncertainties. The study found that elevated CO_2 inhibited MeHg production, with an uncertainty range of 36% to 68% (Supplementary Table T4-1).

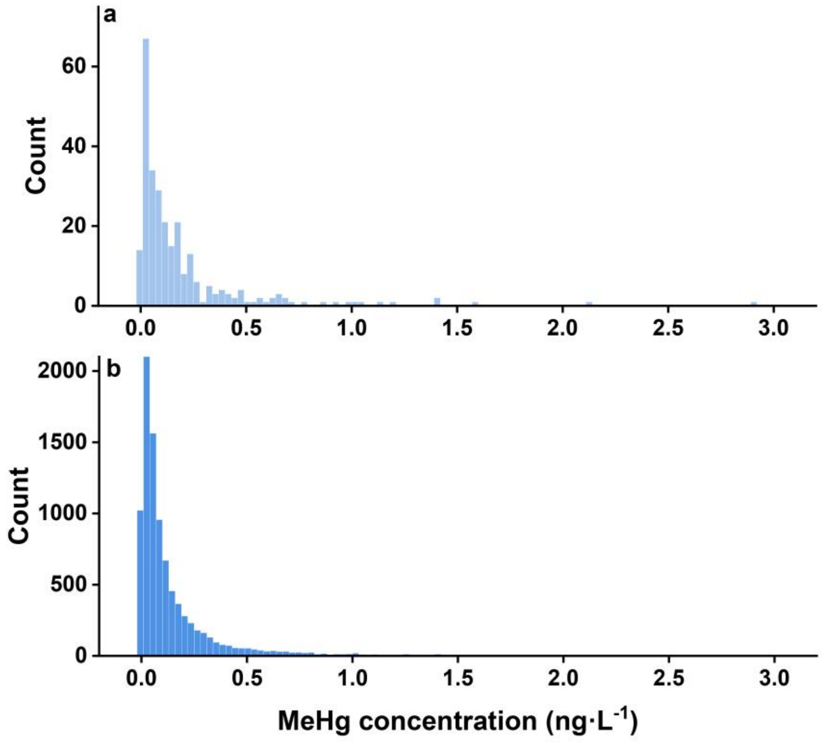

Supplementary Fig. T4-2 The levels of dissolved MeHg in the water from global lakes.

(a) The levels of MeHg collected from previous studies as well as observational data from this study. (b) 10000 MeHg concentrations generated from the lognormal distribution of the reported concentrations.

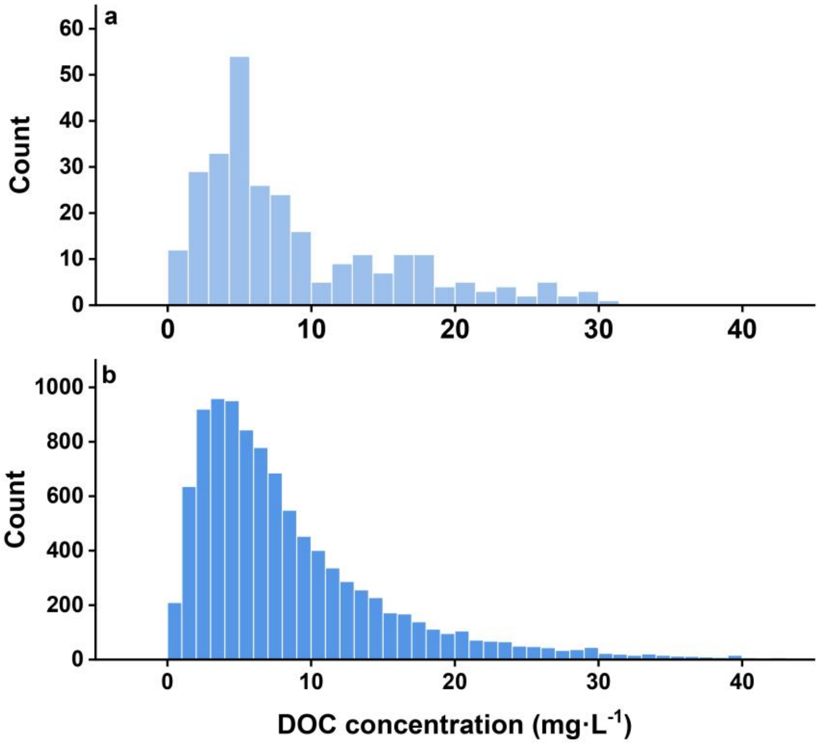

Supplementary Fig. T4-3 The levels of DOC in the water from global lakes. (a) The levels of DOC collected from previous studies as well as observational data from this study. (b) 10000 DOC concentrations generated from the lognormal distribution of the reported concentrations.

Supplementary Table T4-1 Uncertainties of elevated CO_2 inhibited MeHg production.

Parameter	median	average	SD	5% uncertainty	95% uncertainty
%MeHg decreased	43%	47%	12%	36%	68%

Supplementary Text 5 Ignorance of self-stabilization could lead to overestimation of the impacts of climate change on MeHg risks

A recent study used a global ocean model to investigate the effects of climate change on marine Hg cycling, specifically seawater temperature, surface wind speed, sea ice content and light conditions [21]. The study projected a 33% decrease in global average surface ocean MeHg concentrations under the future scenario when compared to present days. Furthermore, there was a notable decrease in chlorophyll and sea ice, resulting in an increase in photo-demethylation potential of MeHg, contributing to reduce MeHg levels in surface ocean water [21].

While for freshwater systems, the higher complexity and more dynamic changes in DOM composition and microbial community structure [22,23], particularly in eutrophic lakes, make it challenging to predict microbe-mediated Hg methylation. These gaps in turn result in significant uncertainties in predicting the risk of MeHg in freshwater lakes in the context of climate change. In this study, we found that elevated CO_2 in the future would inhibit microbial Hg methylation and reduce MeHg levels in lake waters by suppressing the activities and abundances of methanotrophic bacteria (particularly aceticlastic methanogens), particularly in eutrophic lakes. More importantly, such inhibition of microbial MeHg production could be also observed in other freshwater systems (i.e., 79% decrease for pond water and 75% decrease for river water,

Supplementary Fig. 6), implying that elevated CO_2 may mitigate MeHg risk in a wider range, e.g., expanding to the global inland freshwater system. Consequently, elevated CO_2 may partially offset the risk of enhanced MeHg levels in eutrophic freshwaters, which are largely attributed to the massive production of algal organic matter during algal blooms [8]. Further investigation is needed to determine whether these self-stabilizing mechanisms can be extrapolated to oceanic systems or forest systems where Hg cycling is subject to complex impacts of multiple climatic factors [21,24,25].

Supplementary Text 6 Abundances of microbial Hg methylators (Archaea and Deltaproteobacteria) analysis

The production of MeHg in the environment is primarily driven by anaerobic microorganisms including sulfate-reducing bacteria (SRB), iron-reducing bacteria (FeRB), methanogens, and other Firmicutes [26]. Two genes, hgcA and hgcB, have been identified to be essential for microbial Hg methylation [26], using as biomarkers for quantifying the distribution or diversity of Hg-methylating microorganisms in natural environment. By applying publicly available microbial metagenome analysis, phylogenetically diverse hgcA genes have been reported in a wide range of environments [27]. Based on shotgun metagenomic sequencing, the assignments of hgcA genes at the genus level could be obtained. The abundances of the hgcA gene could be quantified by using the clade-specific degenerate primer pairs ORNL-Delta-HgcA and ORNL-Archaea-HgcA for Deltaproteobacterial methylators (e.g., SRB or FeRB) and Archaeal methylators (e.g., methanogens), respectively [28,29].

Total microbial genomic DNA was extracted from water samples using the DNeasy® PowerWater® Kit (QIAGEN, Catalog number: 14900-50-NF, Germany) according to the manufacturer's instructions [30]. The quality and concentration of DNA were determined by 1.0% agarose gel electrophoresis and a NanoDrop2000 spectrophotometer (Thermo Scientific, United States) and kept at -80℃ prior to further use. The abundance of the hgcA gene was

quantified using the clade-specific degenerate primer pairs ORNL-Archaea-HgcA for the Archaeal methylators and ORNL-Delta-HgcA for the Deltaproteobacteria methylators [31], respectively, by amplification of the hgcA sequences from extracted DNA with an iCycler iQ5 thermocycler (Bio-Rad, USA). The primers for Archaea were ORNL-Archaea-HgcA-F (5’-AAYTAYWCNCTSAGYTTYGAYGC-3’) and ORNL-Archaea-HgcA-R (5’-TCDGTCCCRAABGTSCCYTT-3’). Primers of ORNL-Delta-HgcA-F (5’-GCCAACTACAAGMTGASCTWC-3’) and ORNL-Delta-HgcA-R (5’-CCSGCNGCRCACCAGACRTT-3’) were used for Deltaproteobacteria. The isolated DNA was diluted 10-fold and subjected to real-time quantitative PCR (also referred to qPCR) to obtain the abundance of hgcA genes in sediments. Each 25 μL reaction mixture contained 12.5 μL SYBR premix Ex Taq (TaKaRa Bio Inc., Japan), 0.5 μL each of 10 μM forward and reverse primers mentioned above and 2 μL of 10-fold diluted DNA template (1-10 ng). Optimized PCR thermal cycling parameters for Archaeal primers were set as follows: 3 min initial denaturation at 95 °C, 40 cycles of 15 s at 95 °C, 30 s at 50 °C, and 25 s at 55 °C, 4 min at 72 °C, followed by a plate read at 83 °C. As for Deltaproteobacterial primers, the thermal-cycling conditions were set as: 3 min initial denaturation at 95°C, 40 cycles of 15 s at 95°C, 15 s at 50°C, and 15 s at 55°C, 4 min at 72 °C, followed by a plate read at 83°C. Another five replicates, i.e., 25 μL reaction mixtures without the template, were set as negative controls

in the experiments. Besides, PCR amplicons of hgcA genes from Archaea and Deltaproteobacteria were ligated to a pGEMT Easy vector (Promega, USA) and transformed into Escherichia coli JM109 cells (TaKaRa Bio Inc., Japan) according to the manufacturer's protocols, in order to check amplicon sequences and obtain a standard curve for quantification. Then, positive clones containing the target gene insert were sequenced, and the most abundant one was used for plasmid DNA extraction. After measuring the DNA concentration by using a Nanodrop ND-1000 UV-visible spectrophotometer (NanoDrop Co., USA), the purified plasmid DNA was diluted serially in 10-fold steps and subjected to qPCR in triplicate to generate an external standard curve [32].

Supplementary Text 7 Methanogens analysis

The qPCR targeted the mcrA gene encoding the alpha-subunit of methyl coenzyme M reductase (MCR) for the methanogenic communities [33]. The mcrA gene was amplified with primer pairs MLfF (5′-GGTGGTGTMGGATTCACACARTAYGCWACAGC-3′) and MLrR (5′-TTCATTGCRTAGTTWGGRTAGTT-3′) by ABI GeneAmp® PCR System 9700 [34]. The PCR reaction mixture including 10 μL 2 × Pro Taq, 0.8 μL each primer (5 μM), 0.4 μL Fast Pfu polymerase, 10 ng of template DNA, and ddH2O to a final volume of 20 μL. PCR amplification cycling conditions were as follows: initial denaturation at 95 ℃ for 3 min, followed by 27 cycles of denaturing at 95 ℃ for 30 s, annealing at 55 ℃ for 30 s and extension at 72 ℃ for 45 s, and single extension at 72 ℃ for 10 min, and end at 10 ℃. The PCR product was extracted from 2% agarose gel and purified using the PCR Clean-Up Kit (YuHua, Shanghai, China) according to the manufacturer’s instructions and quantified using Qubit 4.0 (Thermo Fisher Scientific, USA). Purified amplicons were pooled in equimolar amounts and paired-end sequenced on an Illumina PE300 platform (Illumina, San Diego, USA) according to the standard protocols by Majorbio Bio-Pharm Technology Co. Ltd. (Shanghai, China).

Raw FASTQ files were de-multiplexed using an in-house perl script, and then quality-filtered by fastp version 0.19.6 [35] and merged by FLASH version 1.2.7 [36] with the following criteria: (i) the reads were truncated at any site

receiving an average quality score of <20 over a 50 bp sliding window, and the truncated reads shorter than 50 bp were discarded, reads containing ambiguous characters were also discarded; (ii) only overlapping sequences longer than 10 bp were assembled according to their overlapped sequence. The maximum mismatch ratio of the overlap region is 0.2. Reads that could not be assembled were discarded; (iii) Samples were distinguished according to the barcode and primers, and the sequence direction was adjusted, exact barcode matching, 2 nucleotide mismatches in primer matching. Then the optimized sequences were clustered into operational taxonomic units (OTUs) using UPARSE 7.0.1090 (http://drive5.com/uparse/) [37,38] with a 97% sequence similarity level. The most abundant sequence for each OTU was selected as a representative sequence. To minimize the effects of sequencing depth on alpha and beta diversity measure, the number of mcrA gene sequences from each sample was rarefied to 25,576, which still yielded an average Good's coverage of 99.09%, respectively. The taxonomy of each OTU representative sequence was analyzed by RDP Classifier version 2.11 (http://sourceforge.net/projects/rdp-classifier/) [39] against the fgr/mcrA_202012 database using a confidence threshold of 0.7.

Supplementary Text 8 Experimental microcosm setup

Experiment A aimed to reveal the general phenomenon of elevated CO_2 on microbial MeHg production in different natural or eutrophic (biomass-amended) lake waters. This was accomplished by comparing the concentrations of dissolved MeHg in water with or without algal biomass under ambient CO_2 (i.e., 420 ppm, "Ambient CO_2") or elevated CO_2 levels (i.e., 650 or 1000 ppm, "Elevated CO_2"). The experimental CO_2 levels were chosen because atmospheric CO_2 levels have historically increased from ~ 280 ppm before the Industrial Revolution to ~420 ppm today, and are projected to rise to ~650 ppm or ~1000 ppm in 2100, corresponding to the intermediate (SSP2-4.5) or high (SSP5-8.5) GHG emissions scenario, as outlined in the Sixth Assessment Report from Intergovernmental Panel on Climate Change (IPCC) [40]. Experiment A was carried on for water collected from all 45 lakes. Briefly, 40 mL of unfiltered water was added to a 50 mL tube (Corning, USA). A level equivalent to 200 $ng \cdot L^{-1}$ of $HgCl_2$ was spiked into the lake water to simulate external Hg sources while relevant to natural conditions (e.g., 20-1000 $ng \cdot L^{-1}$) [41,42]. From each "+Algae" treatment, 0.005 g of dried algal biomass was added into the unfiltered lake water, resulting in approximately 1000 $\mu g \cdot L^{-1}$ chlorophyll *a* level, which is comparable to field observations (e.g., up to 1200 $\mu g \cdot L^{-1}$) in eutrophic lakes during algal blooms [43]. All mixtures were vortex-mixed for 1 min. Tubes were kept open and incubated in incubators at ambient or elevated CO_2 levels.

Ambient CO_2 level (~420 ppm) was achieved by gently aerating the incubator (HP600GS-LED, Wuhan Ruihua Instrument &Equipment Co., Ltd.) with filtered ambient air. Meanwhile, elevated CO_2 levels used calibrated gas mixtures of air and commercially prepared CO_2 gas ensuring target CO_2 levels (approximately 650 or 1000 ppm) and atmospheric O_2 partial pressure (~20%). This design explicitly prevented CO_2-induced anaerobiosis shifts. Dissolved oxygen (DO) measurements confirmed no significant differences ($p > 0.05$) between ambient and elevated CO_2 treatments (1000 ppm) across lake water controls and algal-amended treatments at most time points (Supplementary Fig. T8-1), consistent with microaerophilic conditions in eutrophic lakes where methylators thrive. The CO_2 levels in the incubator were automatically monitored and adjusted in real time withing 5% variability [44]. The experiment was performed in triplicates for each treatment. Tubes were shaken twice per day throughout the incubation. All vials were incubated in the dark at 25 ºC to mimic the prevalent degradation conditions in lakes and preclude MeHg photodegradation. The sampling time point was set at day 7, aligning with changes in bioavailability of AOM that controls the Hg methylating microbes [8]. Ultrapure water was added to the tubes every few days to compensate for water evaporation, ensuring that the final volume was stable at 40 mL. After incubation, the mixtures were centrifuged at 2950 g for 20 min, and the supernatant was collected and filtered for dissolved MeHg assays.

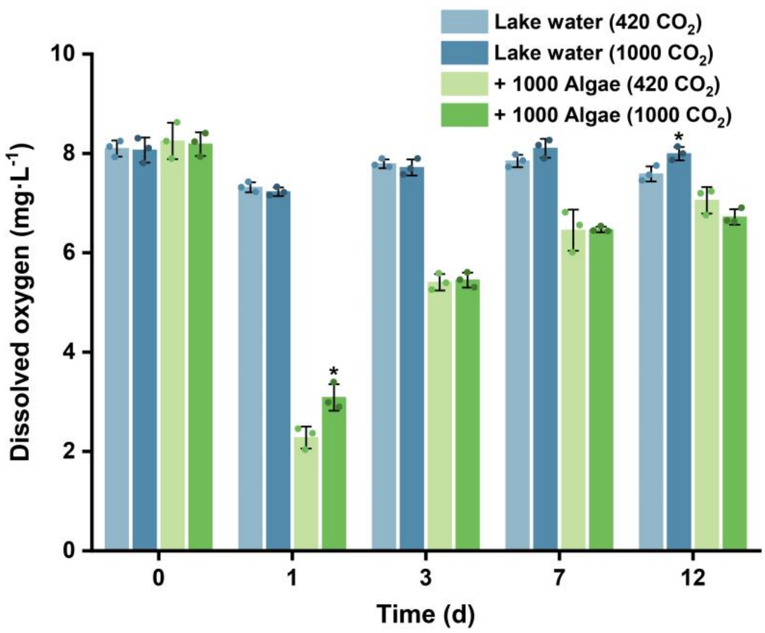

Supplementary Fig. T8-1 Dissolved oxygen (mean ± SD; *n* = 3) in "Lake water" and "+1000 Algae" during incubation under ambient CO2 (420 ppm) or elevated CO2 (1000 ppm) levels.

Experiment B involved the introduction of different amounts of algal biomass to investigate the influence of elevated CO_2 on MeHg production under different eutrophication scenarios. Unfiltered lake water from Chaohu Lake, a large eutrophic system characterized by frequent algal blooms and high AOM, was spiked with $HgCl_2$ and then added with different amounts of algal biomass, i.e., 0 ("Lake water"), 0.001 g ("+200 Algae"), 0.005 g ("+1000 Algae"), and 0.01 g ("+2000 Algae") to simulate different nutrient statuses of lakes [8]. The resulting chlorophyll concentrations were 15.3±1.76 $\mu g \cdot L^{-1}$ for "Lake water" (oligotrophication), 241±19.4 $\mu g \cdot L^{-1}$ for "+200 Algae" (mild eutrophication), 969±20.0 $\mu g \cdot L^{-1}$ for "+1000 Algae" (moderate eutrophication), and 1919±59.0 $\mu g \cdot L^{-1}$ for "+2000 Algae" (severe eutrophication), respectively. All treatments

(e.g., “Ambient CO_2” or “Elevated CO_2”)) and procedures were the same as those employed in Experiment A. Sampling time was scheduled on day 7 and 12 to investigate MeHg production in all treatment conditions.

In addition, Experiment C was used to test for the effects of different CO_2 increase on microbial MeHg production in lake waters. This experiment was carried on for water collected from Chaohu and Yangshan Lake and closely resembled the “+Algae” treatment, as previously described in Experiment A. In this experiment, however, we set CO_2 levels to 420 ppm (referred to as “Ambient CO_2”), 650 ppm, and 1000 ppm (referred to as “Elevated CO_2 (650 or 1000 CO_2)”). These levels align to different scenarios of atmospheric CO_2 increase, as outlined in the IPCC report.

Experiment D was designed to elucidate the fundamental mechanisms underlying the effects of elevated CO_2 on MeHg production over longer time. This was achieved by determining the abundances and activities of microbial methylators and analyzing their interrelationships in response to elevated CO_2 levels. This experiment was conducted using water collected from Chaohu lake and like the “+Algae” treatment in Experiment A mentioned above. Sampling time was set at day 0, 3, 7, 12, 18, 24, 38, and 60 to explore the dynamic characteristics of MeHg production for all treatments. Parallel incubations were established to assess the ambient chemical or biological conditions. On days 3, 7, and 24, the tubes were sealed with silicone rubber for a further 24 h to

determine the daily CH_4 production rates. After 24 h, the tubes were shaken for 1 min to equilibrate the CH_4 between the water and the headspace. Five milliliter gas samples from the headspace was used for CH_4 analyses, to indicate methanogenic activities [45]. The mixtures were then centrifuged at 2950 g for 20 min, and the collected supernatants were filtered to measure dissolved organic matter content (as DOC) on a TOC-Vcph analyzer (Shimazu). Aliquots of filtered water were acidified with trace metal grade HNO_3 and analyzed for the variations in dissolved Fe (indicating iron reduction) and dissolved sulfate levels (indicating sulfate reduction).

Experiment E aimed to investigate whether elevated CO_2 affects the inorganic Hg methylation and MeHg demethylation process. We determined the IHg methylation rate (k_m) and MeHg demethylation rate (k_d) using Hg stable isotope tracers under ambient CO_2 or elevated CO_2 levels. These procedures were similar Experiment A. At each sampling point, i.e., days 3 and 7, ^{202}IHg and Me^{200}Hg$^+$ (sourced from Oak Ridge National Laboratory, TN, USA) was added to all treatment mixtures at concentrations like ambient values. Within 5 min after isotope addition, three sets of triplicates (named "t_0" samples) were frozen at -80 °C for subsequent analysis of k_m and k_d. For each treatment, four replicates fortified with $^{202}HgCl_2$ and Me^{200}Hg$^+$ were incubated for 24 h as the samples of "t_f" at 25 °C. The Hg stable isotope samples were analyzed by species-specific isotope dilution and capillary gas chromatography (Trace GC

Ultra, Thermo Fisher, Waltham, MA, USA) hyphenated to an inductively coupled plasma mass spectrometer (Thermo Scientific, XSeries 2 ICP-MS). Briefly, the mixtures were adjusted into pH=4 and isotopic-enriched $Me^{199}HgCl$ was then added as an internal standard to correct for procedural losses. Subsequently, these different Hg species were ethylated with sodium tetraethyl borate. The values of k_m and k_d were calculated by the following equation [46]:

$$k_m = [Me^{202}Hg^+]_t/([^{202}HgCl_2]_0 \times t)$$

$$k_d = (\ln[Me^{200}Hg^+]_0 - \ln[Me^{200}Hg^+]_t)/t$$

where $[^{202}HgCl_2]_0$ and $[Me^{200}Hg^+]_0$ are the initial amount added into the samples, while $[Me^{200}Hg^+]_t$ and $[Me^{202}Hg^+]_t$ are the final amount after the incubation time (t = 24 h).

Experiment F aimed to investigate the combined effects of warming and elevated CO_2 on MeHg production. This experiment used water collected from Yangshan Lake, following the same setup as the "Lake water" and "+1000 Algae" treatments in Experiment B mentioned above. Incubations were conducted in the dark for 7 days under four conditions: (1) "Control": 25°C and ambient CO_2 (420 ppm); (2) "Warming": 29.4°C (ΔT=4.4°C) and ambient CO_2 (420 ppm); (3) "Elevated CO_2": 25°C and elevated CO_2 (1000 ppm); (4) "Warming × Elevated CO_2": 29.4°C and elevated CO_2 (1000 ppm). Prior to incubation, 200 $ng \cdot L^{-1}$ of $HgCl_2$ was added, and MeHg production was measured on day 7.

Experiment G examined the effects of elevated CO_2 on MeHg production

under different DOM sources and mixing ratios. Soil samples were collected from the riparian zone of Chaohu Lake near the algal sampling site, air-dried, ground, and sieved (0.15 mm). DOM was extracted from soil and algal biomass using surface lake water (soil: water = 1:2, algae: water = 1:100, w/w) in the dark at 25 °C for 12 h, centrifuged (2950 g, 10 min), filtered (0.45 μm PES), and diluted to 20 $mg \cdot L^{-1}$ DOC. To generate mixing gradients, algae-derived (AOM) and soil-derived (SOM) DOM stock solutions were combined at fixed ratios: 100:0 (AOM), 80:20 (M-1), 50:50 (M-2), 20:80 (M-3), and 0:100 (SOM). For the "Lake water" treatment: 40 mL of unfiltered lake water; for each "+DOM" treatment: 20 mL lake water + 20 mL DOM stock solution (final DOC $\approx$ 12.5 $mg \cdot L^{-1}$, comparable to the "+1000 Algae" treatment $\approx$ 13.3 $mg \cdot L^{-1}$). All treatments were spiked with 200 $ng \cdot L^{-1}$ $HgCl_2$ and incubated for 7 days under either ambient (420 ppm) or elevated (1000 ppm) CO_2.

Experiment H evaluated the effects of elevated CO_2 on MeHg production across a gradient of IHg concentrations. This experiment also used Yangshan Lake water, following the design of the "Lake water" and "+1000 Algae" treatments in Experiment B. IHg was added at five concentrations (2, 20, 100, 200 and 500 $ng \cdot L^{-1}$ as $HgCl_2$), and all treatments were incubated for 7 days under ambient (420 ppm) or elevated (1000 ppm) CO_2.

In Experiments B-H, water from Chaohu and Yangshan Lakes was selected to represent a broad range of ecological contexts along the Yangtze

River. Chaohu Lake, a large eutrophic system characterized by frequent algal blooms, exemplifies high AOM conditions and active methanogen-mediated methylation. Yangshan Lake, a suburban lake with relatively low human disturbance, was chosen to capture contrasting trophic states. While the two lakes are highlighted for clarity, the conclusions are supported by data from 45 freshwater lakes across the Yangtze Basin, ensuring broad applicability.

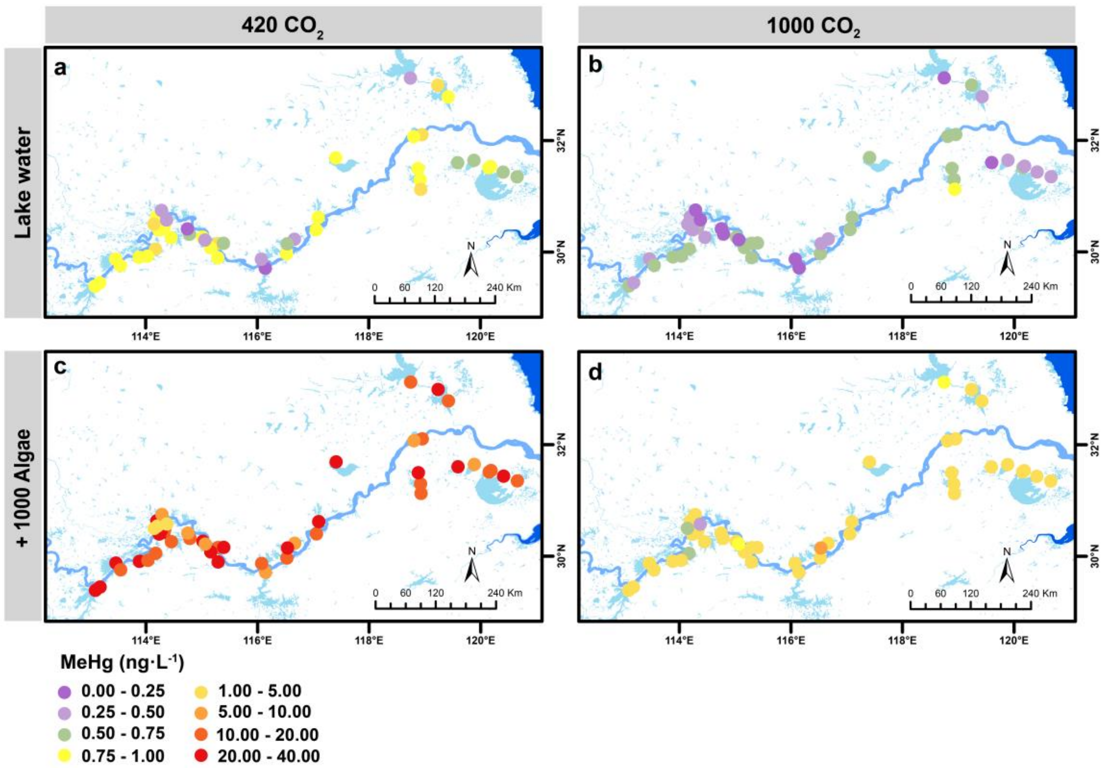

Supplementary Fig. 1 Elevated CO_2 (1000 ppm) decreased the levels of MeHg in water from lakes along the middle and lower reaches of the Yangtze River. (a) the levels of MeHg in “Lake water” groups under 420 ppm CO_2. (b) the levels of MeHg in “Lake water” groups under 1000 ppm CO_2. (c) the levels of MeHg in “+ 1000 Algae” treatments under 420 ppm CO_2. (d) the levels of MeHg in “+ 1000 Algae” treatments under 1000 ppm CO_2. The sampling time was set to Day 7. “+ 1000 Algae”: unfiltered lake water (40 mL) amended with 0.005 g of algal biomass from Chaohu Lake. “Lake water”: unfiltered lake water with no algal addition. The map depicting the middle and lower reaches of the Yangtze River was generated from 1:1,000,000 National Fundamental Geographic Information Data of China (https://www.webmap.cn/commres.do?method=result100W).

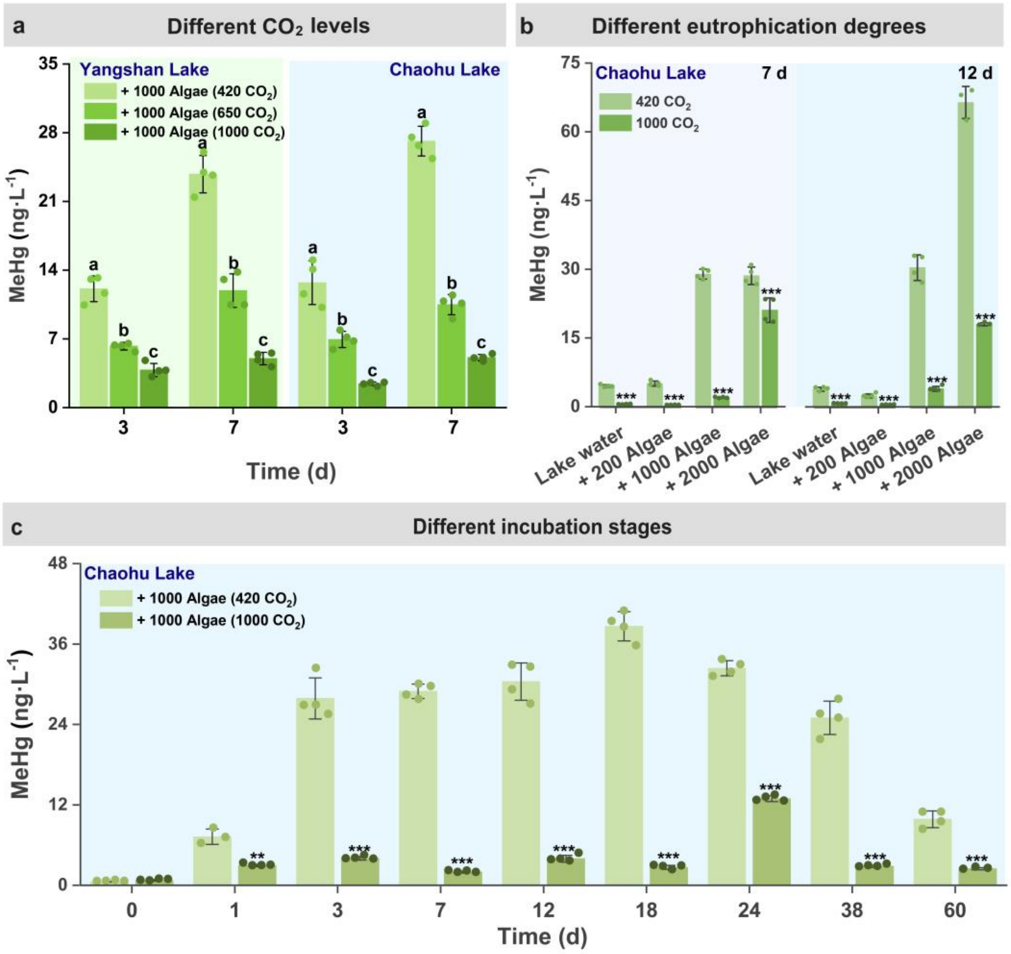

Supplementary Fig. 2 Net MeHg production (mean ± SD; *n* = 3 or 4) in different scenarios under ambient (420 ppm) or elevated CO_2 (1000 ppm) conditions. (a) Different CO_2 levels; (b) Different trophic statuses; (c) Different decomposition stages. "Lake water": unfiltered lake water. "+200 Algae", "+1000 Algae", and "+2000 Algae": unfiltered lake water (40 mL) amended with 0.001, 0.005, and 0.01 g of algal biomass, respectively. Before sampling on "Day 0", all lake water was equilibrated for 4 h with Hg(II). Different lowercase letters above the bars indicate significant differences among different treatments ($p < 0.05$). The asterisk above the bars indicates a significant difference between "420 ppm CO_2" and "1000 ppm CO_2" (*: $p < 0.05$; **: $p < 0.01$; ***: $p < 0.001$; without *: $p > 0.05$).

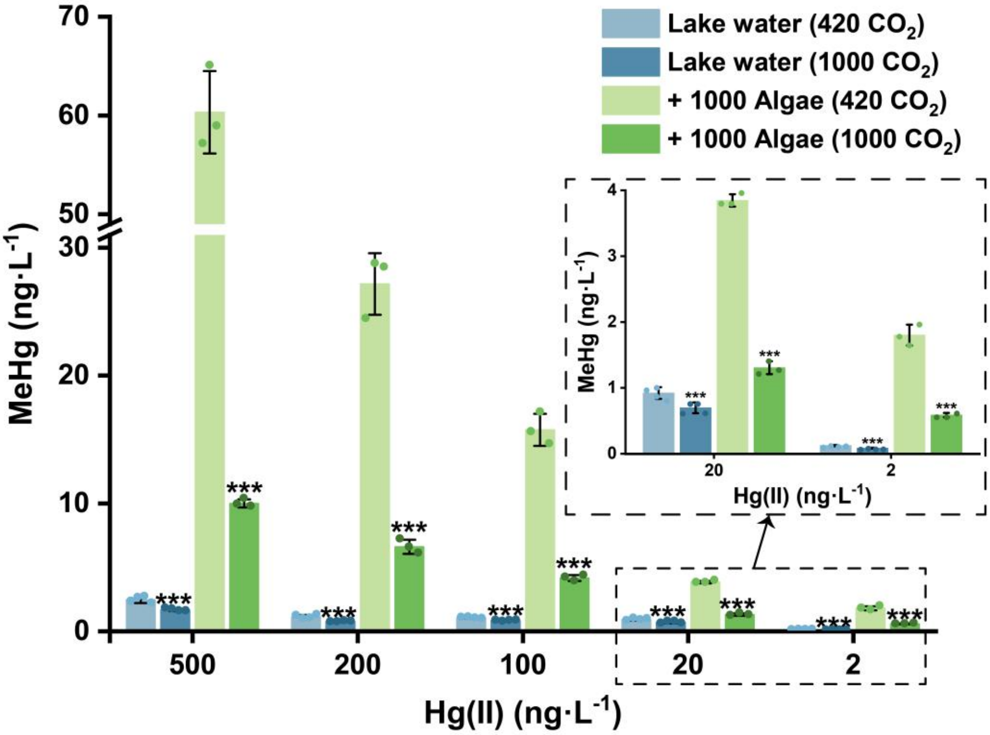

Supplementary Fig. 3 Effect of elevated CO_2 on MeHg production across different IHg concentrations. Levels equivalent to 2, 20, 100, 200 and 500 ng·L^{-1} of $HgCl_2$ were spiked into the lake water, respectively. “Lake water”: unfiltered lake water from Yangshan Lake (located in Nanjing City, China); “+ 1000 Algae”: unfiltered lake water (40 mL) amended with 0.005 g of algal biomass from Chaohu Lake. The asterisk above the bars indicates a significant difference between “420 ppm CO_2” and “1000 ppm CO_2” (*: $p < 0.05$; **: $p < 0.01$; ***: $p < 0.001$; without *: $p > 0.05$). Error bars show standard errors of the mean (n = 3 or 4).

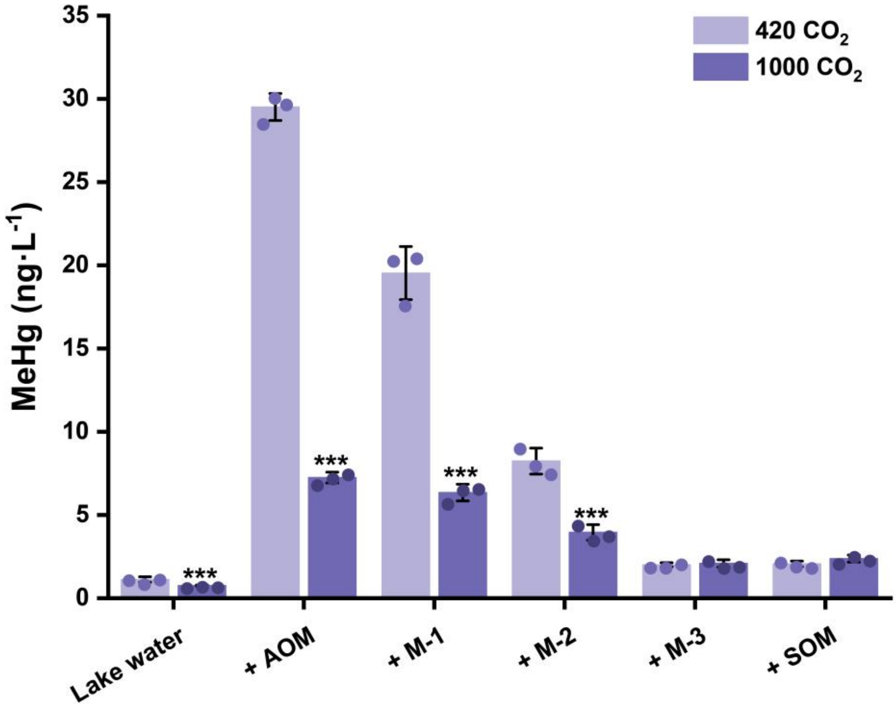

Supplementary Fig. 4 Effects of elevated CO_2 (1000 ppm) on MeHg production (mean ± SD; *n* = 3) compared to ambient CO_2 (420 ppm) under different DOM sources and mixing ratios. Soil samples were collected from the riparian zone of Chaohu Lake. DOM was extracted from soil and algal biomass using water (soil: water = 1:2, algae: water = 1:100, w/w) in the dark at 25 °C for 12 h, centrifuged (2950 g, 10 min), filtered (0.45 μm PES), and diluted to 20 mg·L^{-1} DOC. To generate mixing gradients, algae- (AOM) and soil-derived (SOM) DOM stock solutions were combined at fixed ratios: 100:0 (AOM), 80:20 (M-1), 50:50 (M-2), 20:80 (M-3), and 0:100 (SOM). For the "Lake water": 40 mL of unfiltered lake water; for each "+DOM": 20 mL lake water + 20 mL DOM solution (final DOC ≈ 12.5 mg·L^{-1}, comparable to the "+1000 Algae" treatment ≈ 13.3 mg·L^{-1}). All treatments were incubated for 7 days under either ambient (420 ppm) or elevated (1000 ppm) CO_2. The asterisk above the bars indicates a significant difference between "420 ppm CO_2" and "1000 ppm CO_2" (*: $p < 0.05$; **: $p < 0.01$; ***: $p < 0.001$; without *: $p > 0.05$).

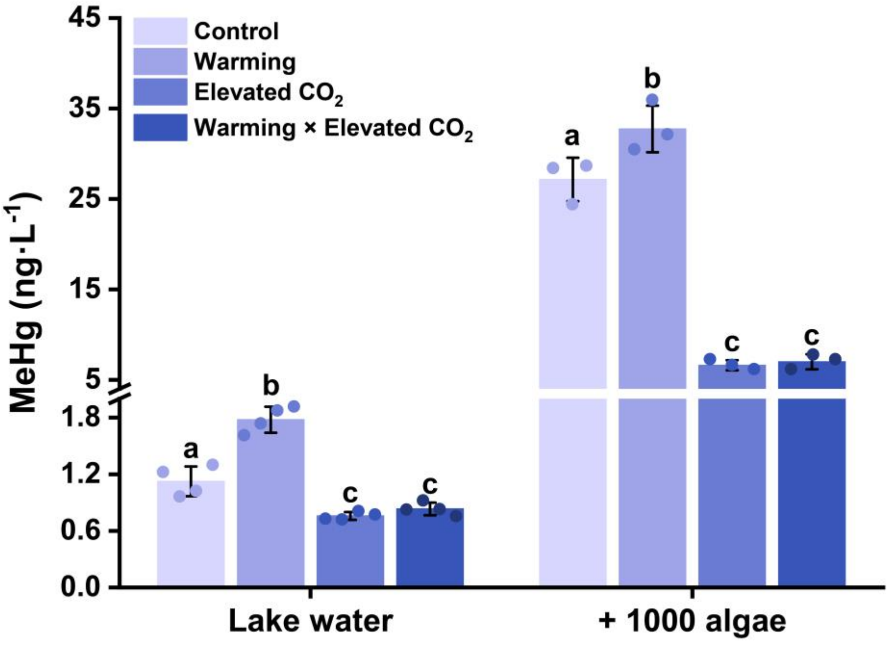

Supplementary Fig. 5 Effects of warming and elevated CO_2 on MeHg production (mean ± SD; *n* = 3 or 4). “Lake water”: unfiltered lake water from Yangshan Lake (Nanjing City, China); “+ 1000 Algae”: unfiltered lake water (40 mL) amended with 0.005 g of algal biomass. “Control”: incubated at 25°C under ambient CO_2 (420 ppm) for 7 days. “Warming”: incubated at 29.4°C (ΔT=4.4°C) and ambient CO_2 (420 ppm). “Elevated CO_2”: incubated at 25°C under elevated CO_2 (1000 ppm); “Warming × Elevated CO_2”: incubated at 29.4°C under elevated CO_2 (1000 ppm). Prior to incubation, 200 ng·L^{-1} of $HgCl_2$ was added into the lake water. Different lowercase letters above the bars indicate significant differences among treatments ($p < 0.05$).

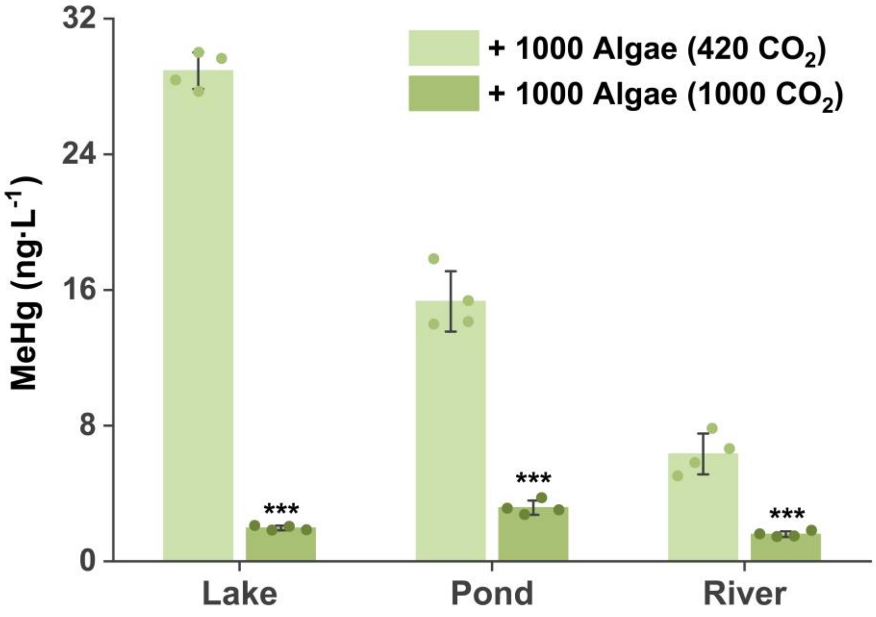

Supplementary Fig. 6 Elevated CO_2 (1000 ppm) decreased the levels of MeHg in lake (93%), pond (79%), and river (75%) water after 7 days of incubation. "+ 1000 Algae": unfiltered lake, river, or pond water (40 mL) amended with 0.005 g of algal biomass from Chaohu Lake. Samples of "Lake", "Pond" and "River" were collected from Chaohu Lake (located in Hefei City, China), Caiyue Pond (located in Nanjing Normal University, Nanjing City, China); and Jiuxiang River (located in Qixia District, Nanjing City, China), respectively. The asterisk above the bars indicates a significant difference between the "420 CO_2" group and the "1000 CO_2" treatment (one-way ANOVA, *, $p < 0.05$; **, $p < 0.01$; ***, $p < 0.001$; without *, $p > 0.05$). Error bars show standard errors of the mean ($n = 4$).

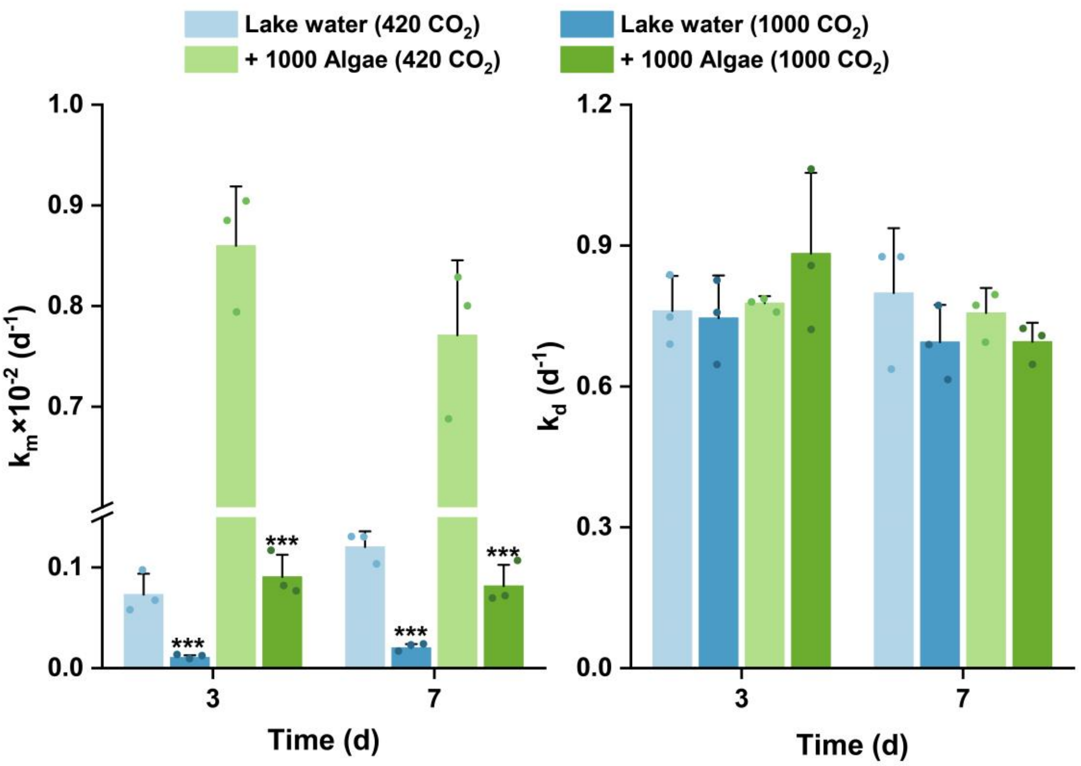

Supplementary Fig. 7 Elevated CO_2 (1000 ppm) had a negligible impact on MeHg demethylation. Effects of elevated CO_2 (1000 ppm) on IHg methylation rates (k_m, d^{-1}) and MeHg demethylation rates (k_d, d^{-1}). Lake water: 40 mL unfiltered lake water; "+ 1000 Algae": unfiltered lake water (40 mL) amended with 0.005 g of algal biomass from Chaohu Lake. Data are represented as mean ± SD of replicate samples (*n*= 3 or 4). The asterisk above the bars indicates a significant difference between the "420 CO_2" group and the "1000 CO_2" treatment (one-way ANOVA, *, $p < 0.05$; **, $p < 0.01$; ***, $p < 0.001$; without *, $p > 0.05$). Error bars show standard errors of the mean ($n = 3$).

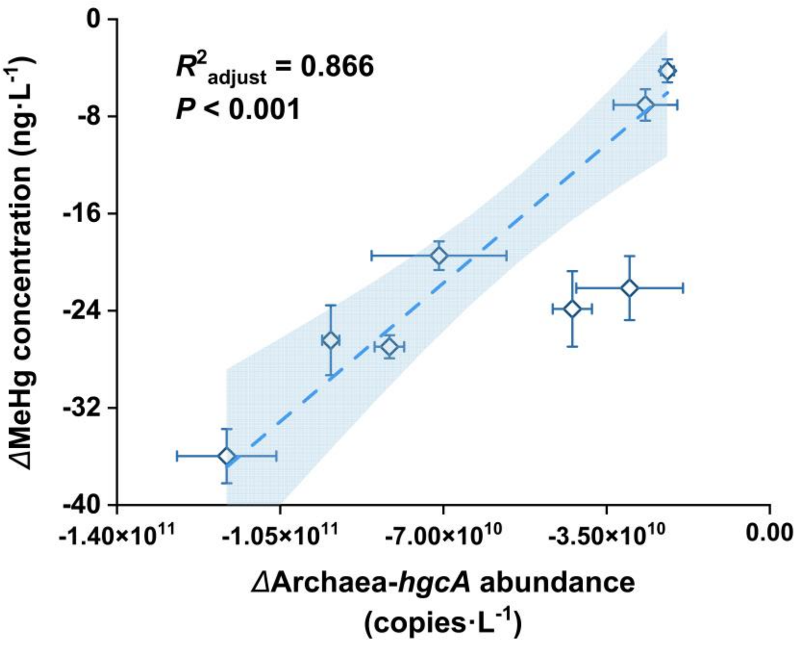

Supplementary Fig. 8 Elevated CO_2-decreased abundance of Archaea-*hgcA* gene was significantly correlated with elevated CO_2-decreased MeHg production. ΔArchaea-*hgcA* abundance："+ 1000 Algae (1000 CO_2)" minus "+ 1000 Algae (420 CO_2); ΔMeHg concentration: "+ 1000 Algae (1000 CO_2)" minus "+ 1000 Algae (420 CO_2). Data of day 0 (i.e., 4 h) was excluded when fitting linear lines due to the limited time for microbial methylators' responses. "+ 1000 Algae": unfiltered lake water (40 mL) amended with 0.005 g of algal biomass from Chaohu Lake. Error bars show standard errors of the mean (n = 3 or 4).

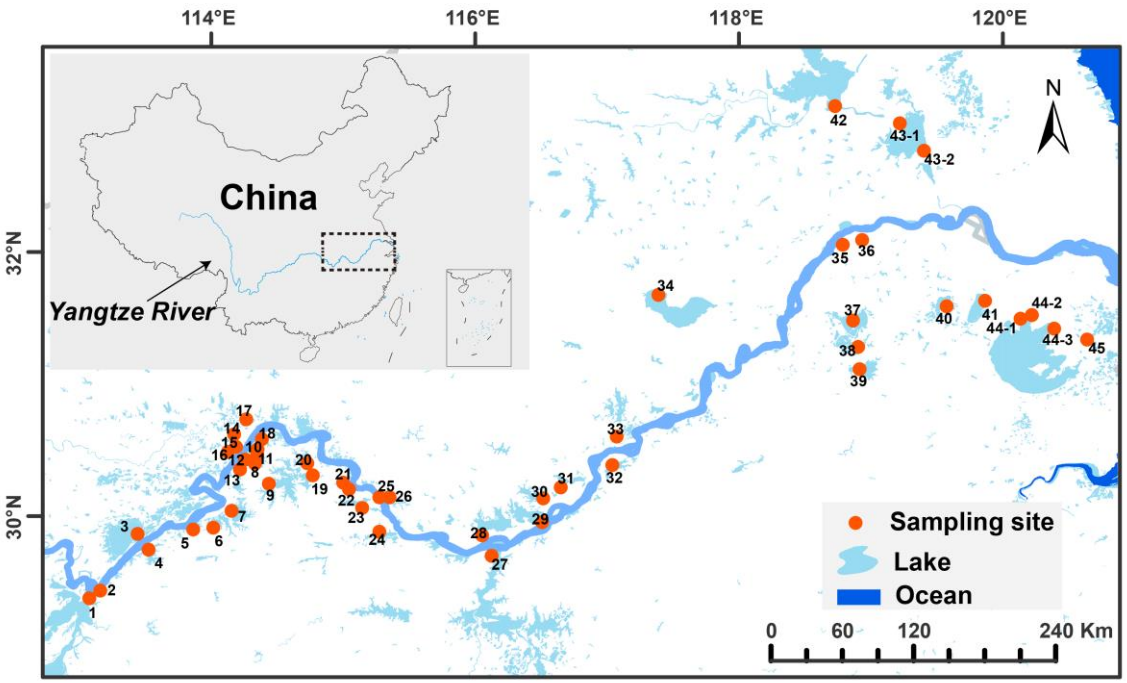

Supplementary Fig. 9 Location of sampling sites from 45 lakes along the middle and lower reaches of the Yangtze River. Specifically, there are 48 sampling sites included. Among them, there are two locations in Gaoyou Lake (No. 43-1 and 43-2), three in Taihu Lake (No. 44-1, 44-2, and 44-3), and one in each of the remaining lakes. The information of sampling sites is shown in Supplementary Table 1. The map of the middle and lower reaches of the Yangtze River was generated using 1:1,000,000 National Fundamental Geographic Information Data of China (https://www.webmap.cn/commres.do?method=result100W). The inset map in the upper-left corner was produced on the basis of a licensed basemap (Map Approval Number: GS(2016)1569) provided by the Ministry of Natural Resources of China (http://bzdt.ch.mnr.gov.cn/).

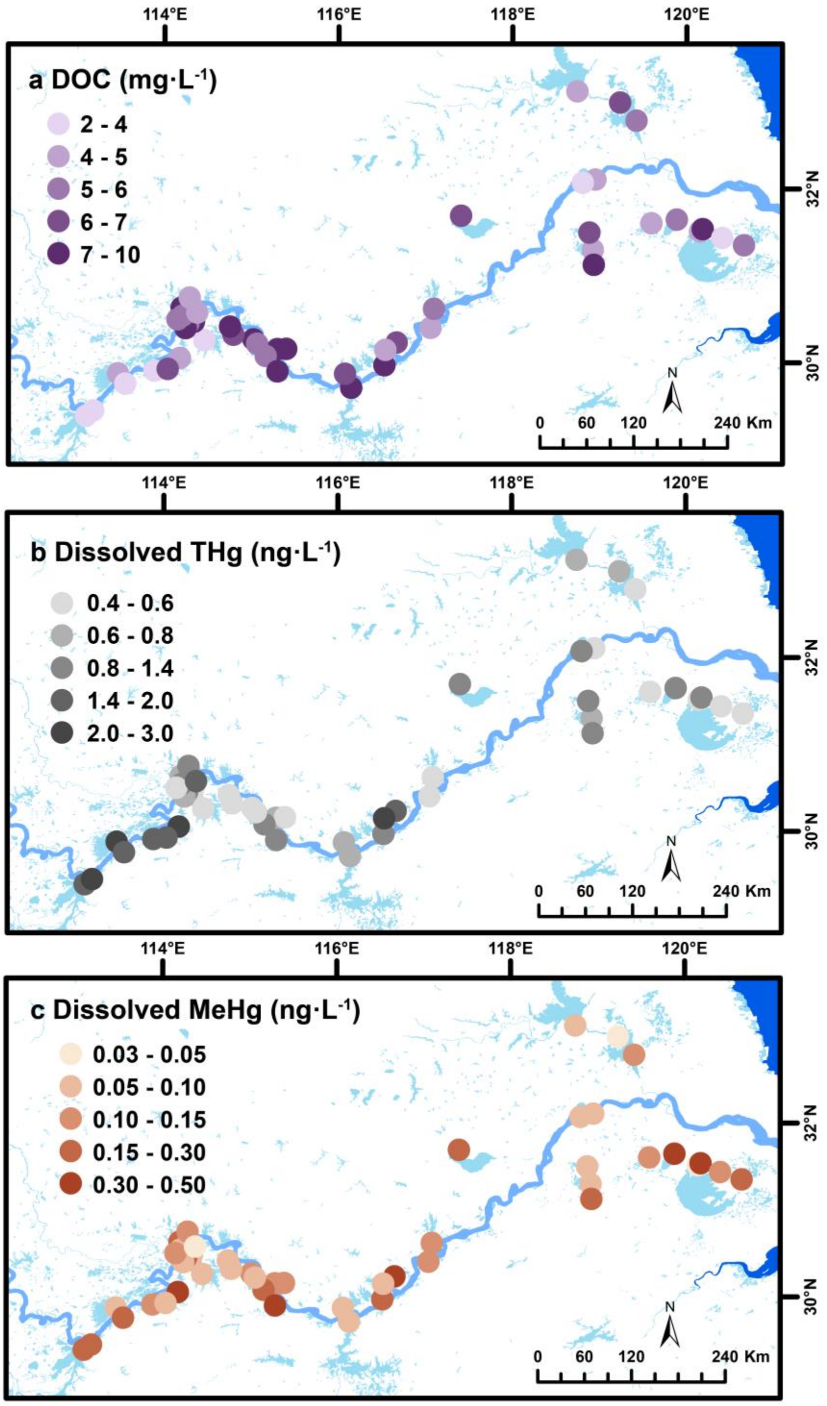

Supplementary Fig. 10 The levels of (a) DOC, (b) dissolved total Hg, and (c)dissolved MeHg in surface water from lakes along the middle and lower reaches of the Yangtze River. The map of the middle and lower reaches of the Yangtze River was generated using 1:1,000,000 National Fundamental Geographic Information Data of China (https://www.webmap.cn/commres.do?method=result100W).

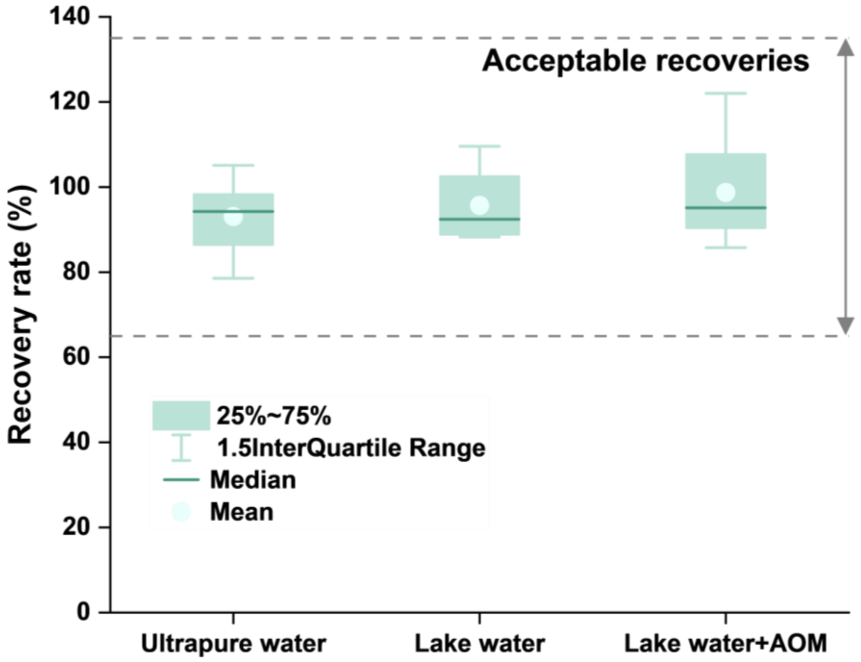

Supplementary Fig. 11 The recovery rates of MeHg standards for ultrapure water blank, lake water, and lake water added with algal biomass.

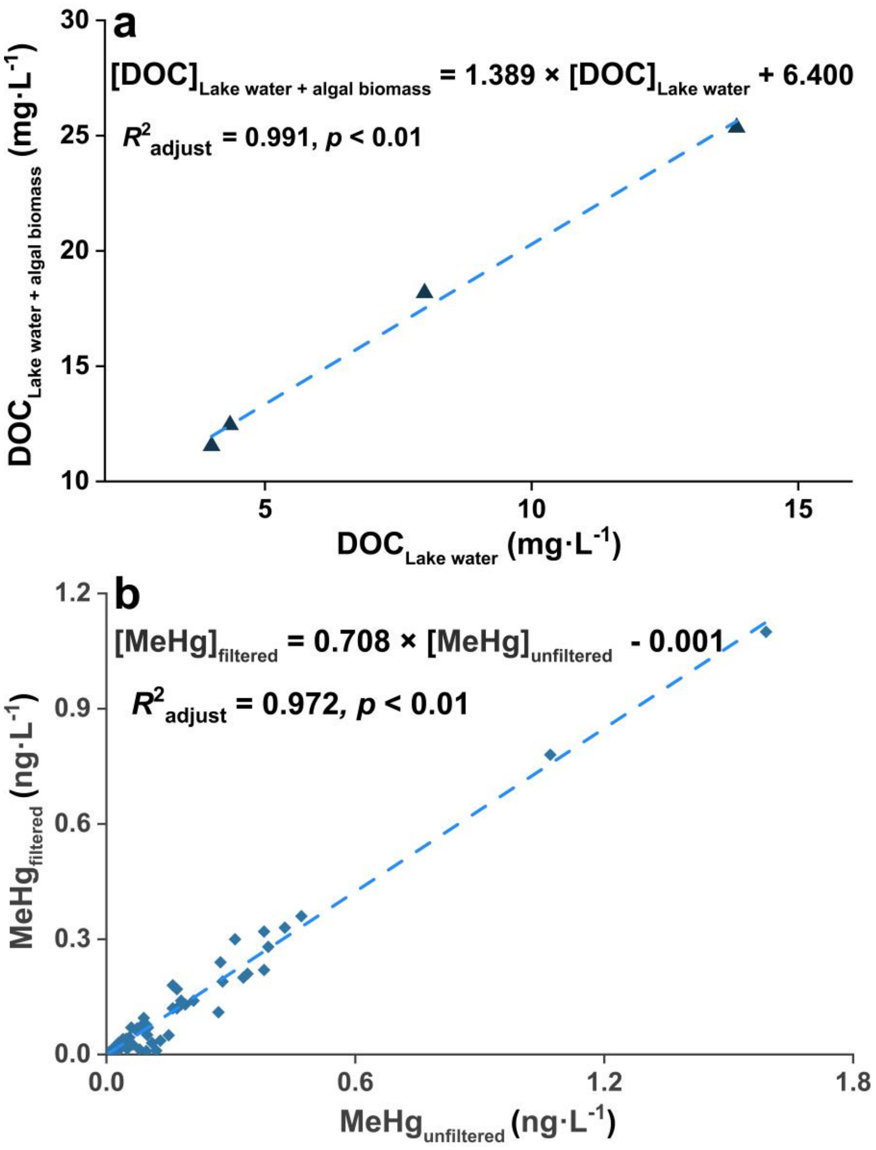

Supplementary Fig. 12 Extrapolation of DOC and MeHg data. (a) Relationship between DOC concentrations in lake water with or without algal organic matter (AOM) input. (b) Relationship between dissolved MeHg concentrations and total MeHg concentrations in lake water.

Supplementary Table 1 The basic information of lakes along the middle and lower reaches of the Yangtze River

No.	Name	latitude/°N	longitude/°E	Lake area/km^2	Mean depth/m	TN^a/mg·L^{-1}	TP^a/mg·L^{-1}	Reference
1	Dongting Lake	29.395	113.090	2432	6.4	2.036	0.041	47
2	Bajiao Lake	29.452	113.173	NA[b]	NA	0.646	0.035	NA
3	Honghu Lake	29.883	113.458	344	1.9	0.238	0.032	47
4	Huanggai Lake	29.764	113.542	86	4.2	1.041	0.035	47
5	Miquan Lake	29.915	113.878	NA	NA	3.018	0.078	NA
6	Xiliang Lake	29.930	114.031	72	1.9	2.946	0.086	47
7	Futou Lake	30.056	114.171	141.2	2.3	0.250	0.033	48
8	Tangxun Lake	30.430	114.318	44.8	2.3	1.185	0.055	48
9	Liangzi Lake	30.263	114.451	349.8	3	1.808	0.026	48
10	Nanhu Lake	30.496	114.337	8	2.4	1.604	0.072	49
11	Yezhi Lake	30.474	114.332	1.6	NA	1.688	0.029	50
12	Huangjia Lake	30.457	114.269	6.8	1.8	4.384	0.064	48
13	Qingling Lake	30.402	114.234	7.2	1.8	2.467	0.040	48
14	Jinyin Lake	30.638	114.190	8.2	NA	1.700	0.068	51
15	Moshui Lake	30.544	114.203	2.7	2.1	2.778	0.053	49
16	Houguan Lake	30.504	114.143	10.1	2.8	4.288	0.209	49
17	Houhu Lake	30.751	114.282	16.1	1.14	3.880	0.179	50
18	Donghu Lake	30.579	114.371	34.2	4	1.592	0.044	48
19	Sanshan Lake	30.326	114.785	23.9	2.6	NA	NA	48
20	Wusi Lake	30.420	114.745	NA	NA	NA	NA	NA
21	Huama Lake	30.271	115.015	10	2.3	NA	NA	48
22	Cihu Lake	30.223	115.060	10	1.5	0.740	0.090	52
23	Daye Lake	30.082	115.158	73.6	4.1	0.730	0.070	52
24	Wanghu Lake	29.902	115.290	42.3	3.7	1.160	0.270	52

25	Chixi Lake	30.160	115.290	NA	NA	NA	NA	NA
26	Chidong Lake	30.161	115.392	26.8	5.8	1.330	0.241	[53]
27	Poyang Lake	29.718	116.141	3192	7.6	1.067	0.040	[53]
28	Longgan Lake	29.873	116.069	295.5	3	0.770	0.040	[52]
29	Huanghu Lake	29.974	116.524	12	2.8	NA	NA	[47]
30	Bohu Lake	30.151	116.534	180	4.4	0.550	0.021	[53]
31	Wuchang Lake	30.237	116.665	107.5	3.9	0.630	0.030	[48]
32	Shengjin Lake	30.406	117.055	95.9	5	0.740	0.021	[53]
33	Pogang Lake	30.622	117.091	52.1	2.5	1.900	0.220	[52]
34	Chaohu Lake	31.695	117.406	788	2.5	0.930	0.040	[52]
35	Xuanwu Lake	32.073	118.802	3.7	1.1	NA	NA	[54]
36	Yangshan Lake	32.110	118.950	NA	NA	NA	NA	NA
37	Shijou Lake	31.500	118.880	214.3	5.3	1.210	0.010	[52]
38	Nanyi Lake	31.130	118.930	197.8	4.4	2.340	0.020	[52]
39	Gucheng Lake	31.300	118.920	31.2	4.3	0.890	0.016	[53]
40	Changdang Lake	31.606	119.592	88	1.1	1.185	0.258	[47]
41	Gehu Lake	31.647	119.883	147	1.2	2.483	0.275	[47]
42	Hongze Lake	33.124	118.743	2069	1.5	0.492	0.169	[54]
43-1	Gaoyou Lake1	32.993	119.236	674.7	1.4	1.182	0.051	[55]
43-2	Gaoyou Lake2	32.788	119.421	674.7	1.4	0.611	0.116	[55]
44-1	Taihu Lake1	31.514	120.151	2444.8	3.1	0.854	0.080	[48]
44-2	Taihu Lake2	31.540	120.185	2444.8	3.1	3.937	0.360	[48]
44-3	Taihu Lake3	31.439	120.407	2444.8	3.1	0.663	0.072	[48]
45	Yangcheng Lake	31.355	120.657	119	1.4	2.041	0.340	[47]

[a] The data for No.1-18 and No.40-48 were measured, while No.19-39 were from literature sources.

[b] N/A: not applicable.

Supplementary Table 2 Treatments in the microcosm experiments

Objectives	Experiments	Abbreviation	Treatments[b]	Sampling time	Parameters
Exploring the effects of elevated CO_2 on microbial Hg methylation	A: 48 lake water with or without algal biomass addition under ambient or elevated CO_2 levels[a]	Lake water (420 CO_2)<br>Lake water (1000 CO_2)<br>+ 1000 Algae (420 CO_2)<br>+ 1000 Algae (1000 CO_2)	40 mL unfiltered lake water (420 ppm CO_2)<br>40 mL unfiltered lake water (1000 ppm CO_2)<br>Lake water + 0.005 g algal biomass (420 ppm CO_2)<br>Lake water + 0.005 g algal biomass (1000 ppm CO_2)	Day 7	Dissolved MeHg
	B: Algal biomass addition within different algal densities for Chaohu Lake water[c]	Lake water (420 CO_2)<br>Lake water (1000 CO_2)<br>+ 200 Algae (420 CO_2)<br>+ 200 Algae (1000 CO_2)<br>+ 1000 Algae (420 CO_2)<br>+ 1000 Algae (1000 CO_2)<br>+ 2000 Algae (420 CO_2)<br>+ 2000 Algae (1000 CO_2)	40 mL unfiltered lake water (420 ppm CO_2)<br>40 mL unfiltered lake water (1000 ppm CO_2)<br>Lake water + 0.001 g algal biomass (420 ppm CO_2)<br>Lake water + 0.001 g algal biomass (1000 ppm CO_2)<br>Lake water + 0.005 g algal biomass (420 ppm CO_2)<br>Lake water + 0.005 g algal biomass (1000 ppm CO_2)<br>Lake water + 0.01 g algal biomass (420 ppm CO_2)<br>Lake water + 0.01 g algal biomass (1000 ppm CO_2)	Day 7 and 12	Dissolved MeHg
	C: Elevated CO_2 within different levels for Chaohu and Yangshan Lake water	+ 1000 Algae (420 CO_2)<br>+ 1000 Algae (650 CO_2)<br>+ 1000 Algae (1000 CO_2)	Lake water + 0.005 g algal biomass (420 ppm CO_2)<br>Lake water + 0.005 g algal biomass (650 ppm CO_2)<br>Lake water + 0.005 g algal biomass (1000 ppm CO_2)	Day 3 and 7	Dissolved MeHg
Elucidating the mechanisms of elevated CO_2-impacted MeHg production	D: Algal biomass addition at different stages of algae decomposition under ambient or elevated CO_2 levels	Lake water (420 CO_2)<br>Lake water (1000 CO_2)<br>+ 1000 Algae (420 CO_2)<br>+ 1000 Algae (1000 CO_2)	40 mL unfiltered lake water (420 ppm CO_2)<br>40 mL unfiltered lake water (1000 ppm CO_2)<br>Lake water + 0.005 g algal biomass (420 ppm CO_2)<br>Lake water + 0.005 g algal biomass (1000 ppm CO_2)	Day 0 (4 h), 3, 7, 12, 18, 24, 38 and 60	Dissolved MeHg, Fe, sulfate, CH_4 production, methanogens community, Archaea- and Deltaproteobacteria-*hgcA* gene abundance

	E: Quantifying the methylation rate (k_m) and demethylation rate (k_d) under elevated CO_2 [e]	Lake water (420 CO_2)<br>Lake water (1000 CO_2)<br>+ 1000 Algae (420 CO_2)<br>+ 1000 Algae (1000 CO_2)	40 mL unfiltered lake water (420 ppm CO_2)<br>40 mL unfiltered lake water (1000 ppm CO_2)<br>Lake water + 0.005 g algal biomass (420 ppm CO_2)<br>Lake water + 0.005 g algal biomass (1000 ppm CO_2)	Day 3 and 7	k_m and k_d
Investigating the effects of warming and elevated CO_2 on MeHg production	F: Quantifying net MeHg production under warming and elevated CO_2 conditions	Control	40 mL unfiltered lake water with or without 0.005 g algal biomass (420 ppm CO_2 & 25°C)	Day 7	Dissolved MeHg
		Warming	40 mL unfiltered lake water with or without 0.005 g algal biomass (420 ppm CO_2 & 29.4°C)		
		Elevated CO_2	40 mL unfiltered lake water with or without 0.005 g algal biomass (1000 ppm CO_2 & 25°C)		
		Warming × Elevated CO_2	40 mL unfiltered lake water with or without 0.005 g algal biomass (1000 ppm CO_2 & 29.4°C)		
Investigating the effects of elevated CO_2 on MeHg production under different DOM sources and mixing ratios	G: DOM extracted from soil and agal biomass addition within different ratios for Lake water under ambient or elevated CO_2 levels	Lake water	40 mL unfiltered lake water (420 or 1000 ppm CO_2)	Day 7	Dissolved MeHg
		+ AOM	20 mL lake water + 20 mL AOM solution (420 or 1000 ppm CO_2)		
		+ M-1	20 mL lake water + 16 mL AOM + 4 mL Soil-derived DOM solution (420 or 1000 ppm CO_2)		
		+ M-2	20 mL lake water + 10 mL AOM + 10 mL Soil-derived DOM solution (420 or 1000 ppm CO_2)		
		+ M-3	20 mL lake water + 4 mL AOM + 16 mL Soil-derived DOM solution (420 or 1000 ppm CO_2)		
		+ SOM	20 mL lake water + 20 mL Soil-derived DOM solution (420 or 1000 ppm CO_2)		
Investigating the effects of	H: Spiking Levels equivalent to 2, 20, 100,	Lake water (420 CO_2)<br>Lake water (1000 CO_2)	40 mL unfiltered lake water (420 ppm CO_2)<br>40 mL unfiltered lake water (1000 ppm CO_2)	Day 7	Dissolved MeHg

elevated CO_2 on MeHg production across different IHg levels	200 and 500 ng·L^{-1} of $HgCl_2$	+ 1000 Algae (420 CO_2)<br>+ 1000 Algae (1000 CO_2	Lake water + 0.005 g algal biomass (420 ppm CO_2)<br>Lake water + 0.005 g algal biomass (1000 ppm CO_2)

[a] These lake water samples were all collected from the middle and lower reaches of the Yangtze River, and their information is shown in Supplementary Table 1.

[b] Mercury was spiked in our microcosm experiments, simulating exogenous Hg input into lake systems, e.g., via runoff or atmospheric deposition. The spiked Hg level (equivalent to 200 ng·L^{-1} in Experiment A, B, C, D, E, F & G) was in the range of Hg concentrations reported in these selected lake water, e.g., 50-1050 ng·L^{-1} in Chaohu Lake. Algal biomass used in all Experiments was taken from Chaohu Lake as an example to elucidate the mechanisms of Elevated CO_2-impacted microbial Hg methylation in eutrophic lakes.

[c] The treatments of "+ 200 Algae" to "+ 2000 Algae" represent different amounts of algal biomass input. The levels of Chl *a* in Experiment B on day 0 of 'Lake water', '+ 200 Algae', '+ 1000 Algae', and '+ 2000 Algae' treatments were 15.3 ± 1.76, 241 ± 19.4, 969 ± 20.0, and 1919 ± 59.0 μg·L^{-1}, respectively.

[d] MeHg was spiked into all treatments with a final MeHg concentration of 10 ng·L^{-1}.

[e] For each treatment in Experiment E-2, four slurry replicates were spiked with enriched ^{200}MeHg at concentrations similar to ambient values at each sampling point.

Supplementary Table 3 Data used in the multiple linear regression analysis

No.[a]	DOC/mg·L^{-1}	Dissolved MeHg/ng·L^{-1}	Reduction rate	N-Reduction rate [b]
1	2.055	0.939	0.402	0.402
2	4.716	0.862	0.474	0.474
3	2.774	0.834	0.434	0.434
4	4.115	1.073	0.363	0.363
5	3.649	0.889	0.323	0.323
6	6.301	0.902	0.267	0.267
7	3.395	0.922	0.386	0.386
8	4.600	0.849	0.402	0.402
9	3.483	0.784	0.398	0.398
10	4.039	0.813	0.389	0.389
11	7.788	0.846	0.487	0.487
12	5.549	0.852	0.379	0.379
13	8.149	0.836	0.437	0.437
14	8.254	0.856	0.496	0.496
15	5.886	0.808	0.514	0.514
16	5.753	1.019	0.572	0.572
17	4.741	0.479	0.528	0.528
18	4.462	0.347	0.485	0.485
19	4.522	0.452	0.452	0.452
20	6.297	1.070	0.525	0.525
21	5.102	0.947	0.504	0.504
22	4.448	0.934	0.448	0.448
23	8.975	0.756	0.441	0.441
24	2.799	0.703	0.554	0.554
25	5.892	0.611	0.550	0.550
26	5.215	0.643	0.526	0.526
27	4.371	0.538	0.578	0.578
28	4.578	0.920	0.386	0.386
29	8.897	1.099	0.295	0.295
30	6.406	0.988	0.415	0.415
31	4.949	1.149	0.465	0.465
32	8.852	1.085	0.395	0.395
33	7.587	0.744	0.284	0.284
34	5.546	0.894	0.286	0.286
35	8.337	0.854	0.168	0.168
36	9.420	0.788	0.253	0.253
37	6.182	0.616	0.603	0.603
38	5.248	0.328	0.282	0.282
39	7.483	0.229	0.433	0.433
40	8.611	0.225	0.247	0.247
41	6.010	0.295	0.225	0.225

42	6.918	0.471	0.267	0.267
43	7.711	0.805	0.238	0.238
44	4.450	0.738	0.419	0.419
45	6.368	0.796	0.141	0.141
46	4.934	0.888	0.356	0.356
47	5.233	0.894	0.416	0.416
48	3.843	0.943	0.318	**0.318**
49	9.254	35.865	0.936	**0.838**
50	12.949	39.297	0.892	**0.798**
51	10.253	30.367	0.935	**0.837**
52	12.115	13.161	0.944	**0.845**
53	11.468	21.810	0.880	**0.788**
54	15.151	10.903	0.730	**0.653**
55	11.115	12.714	0.843	**0.755**
56	12.788	39.011	0.948	**0.848**
57	11.238	18.636	0.931	**0.833**
58	12.009	23.330	0.927	**0.830**
59	17.216	18.891	0.917	**0.821**
60	14.107	22.180	0.934	**0.836**
61	17.718	24.576	0.905	**0.810**
62	17.864	23.027	0.914	**0.817**
63	14.575	2.877	0.544	**0.486**
64	14.390	1.904	0.656	**0.587**
65	12.985	7.425	0.851	**0.761**
66	12.597	2.424	0.798	**0.714**
67	12.681	11.245	0.915	**0.819**
68	15.146	23.711	0.912	**0.816**
69	13.486	18.137	0.873	**0.782**
70	12.578	15.161	0.827	**0.740**
71	18.865	18.462	0.819	**0.733**
72	10.288	26.538	0.814	**0.729**
73	14.583	13.358	0.817	**0.731**
74	13.643	9.541	0.699	**0.625**
75	12.471	21.715	0.919	**0.822**
76	12.758	18.811	0.923	**0.826**
77	18.757	14.620	0.829	**0.741**
78	15.297	21.647	0.913	**0.817**
79	13.274	15.906	0.894	**0.800**
80	18.694	16.423	0.858	**0.768**
81	16.937	24.433	0.958	**0.858**
82	14.102	24.044	0.915	**0.819**
83	17.978	24.323	0.899	**0.804**
84	19.483	21.409	0.847	**0.758**
85	14.986	18.101	0.868	**0.777**
86	13.689	5.844	0.865	**0.774**

87	16.792	6.841	0.820	0.733
88	18.359	7.910	0.838	0.750
89	14.747	16.998	0.789	0.706
90	16.008	8.317	0.687	0.614
91	17.109	16.862	0.850	0.761
92	12.580	26.090	0.694	0.621
93	15.244	28.929	0.932	0.834
94	13.253	19.332	0.876	0.784
95	13.668	24.322	0.887	0.794
96	11.737	7.472	0.833	0.746
97	13.844	4.493	0.888	**0.888**
98	25.354	28.929	0.932	**0.834**

[a] 1-48 and 49-96 represented the results for the lake water control group and "+Algae" treatment group along the middle and lower reaches of the Yangtze River. 97-98 represented the results for Chaohu Lake water and its "+ algae" treatment group from the mechanism investigation experiment. The DOC in "+ Algae" treatments can be estimated as: $[DOC]_{\text{Lake water + algal biomass}}$ = (1.389 ± 0.075) × $[DOC]_{\text{lake water}}$ + (6.4 ± 0.638) (r^2_{adjust} = 0.991, p = 0.003, see Supplementary, Fig. 12).

[b] 49-96 and 98 represented the values of the inhibition rates of MeHg net production in the "+Algae" treatments used in the model after normalization according to the "M1/AOM" ratio (i.e., 89.5%, where M1 represents the mixture of 20% terrestrial organic matter and 80% AOM)

Supplementary Data 1. Data sets of observational DOC and MeHg concentrations in global lakes collected from literature. The publications spanning the period from 2000 to 2023 that the documented MeHg and DOC levels in lake water were retrieved in Web of Science™ on June 6, 2023. From the search results of 201 scientific papers, 49 articles containing 229 samples were obtained for analyses. The document was attached as an Excel file.

Reference

1 Lei, P. *et al.* Algal organic matter inhibits methylmercury photodegradation in eutrophic lake water: A dynamic study. *Science of The Total Environment* **899**, 165661 (2023). https://doi.org:https://doi.org/10.1016/j.scitotenv.2023.165661

2 Eriksson, T., Öquist, M. G. & Nilsson, M. B. Production and oxidation of methane in a boreal mire after a decade of increased temperature and nitrogen and sulfur deposition. *Global Change Biology* **16**, 2130-2144 (2010). https://doi.org:10.1111/j.1365-2486.2009.02097.x

3 Holm, S. *et al.* Methanogenic response to long-term permafrost thaw is determined by paleoenvironment. *FEMS Microbiology Ecology* **96**, fiaa021 (2020). https://doi.org:10.1093/femsec/fiaa021

4 Lei, P. *et al.* Response of mercury methylation to algal bloom decomposition or elevated CO_2 in surface sediments from the East China Sea. *Environmental Pollution* **383**, 126786 (2025).

5 Lyon, B. F., Ambrose, R., Rice, G. & Maxwell, C. J. Calculation of soil-water and benthic sediment partition coefficients for mercury. *Chemosphere* **35**, 791-808 (1997). https://doi.org:https://doi.org/10.1016/S0045-6535(97)00200-2

6 Li, Y. & Cai, Y. Progress in the study of mercury methylation and demethylation in aquatic environments. *Chinese Science Bulletin* **58**, 177-185 (2013). https://doi.org:10.1007/s11434-012-5416-4

7 Wu, Z. Y. *et al.* Differential response of Hg-methylating and MeHg-demethylating microbiomes to dissolved organic matter components in eutrophic lake water. *Journal of Hazardous Materials* **465**, 133298 (2024). https://doi.org:10.1016/j.jhazmat.2023.133298

8 Lei, P. *et al.* Algal organic matter drives methanogen-mediated methylmercury production in water from eutrophic shallow lakes. *Environmental Science & Technology* **55**, 10811-10820 (2021). https://doi.org:10.1021/acs.est.0c08395

9 Zhang, Y., Dutkiewicz, S. & Sunderland, E. M. Impacts of climate change on methylmercury formation and bioaccumulation in the 21st century ocean. *One Earth* **4**, 279-288 (2021). https://doi.org:https://doi.org/10.1016/j.oneear.2021.01.005

10 Kakuk, B. *et al.* Early response of methanogenic archaea to H2 as evaluated by metagenomics and metatranscriptomics. *Microbial Cell Factories* **20**, 127 (2021). https://doi.org:10.1186/s12934-021-01618-y

11 Conrad, R. Importance of hydrogenotrophic, aceticlastic and methylotrophic methanogenesis for methane production in terrestrial, aquatic and other anoxic environments: A mini review. *Pedosphere* **30**, 25-39 (2020). https://doi.org:10.1016/s1002-0160(18)60052-9

12 Demirel, B. & Scherer, P. The roles of acetotrophic and hydrogenotrophic methanogens during anaerobic conversion of biomass to methane: a review. *Reviews in Environmental Science and Bio/Technology* **7**, 173-190 (2008). https://doi.org:10.1007/s11157-008-9131-1

13 Szuhaj, M. *et al.* Conversion of H2 and CO2 to CH4 and acetate in fed-batch biogas

reactors by mixed biogas community: a novel route for the power-to-gas concept. *Biotechnology for Biofuels* **9**, 102 (2016). https://doi.org:10.1186/s13068-016-0515-0
14 Zhao, Y. P. *et al.* High sulfide production induced by algae decomposition and its potential stimulation to phosphorus mobility in sediment. *Science of the Total Environment* **650**, 163-172 (2019). https://doi.org:10.1016/j.scitotenv.2018.09.010
15 Tong, H., Hu, M., Li, F. B., Liu, C. S. & Chen, M. J. Biochar enhances the microbial and chemical transformation of pentachlorophenol in paddy soil. *Soil Biology & Biochemistry* **70**, 142-150 (2014). https://doi.org:10.1016/j.soilbio.2013.12.012
16 Wang, J. X. *et al.* Mobilization, methylation, and demethylation of mercury in a paddy soil under systematic redox changes. *Environmental Science & Technology* **55**, 10133-10141 (2021). https://doi.org:10.1021/acs.est.0c07321
17 Hrabik, T. R. & Watras, C. J. Recent declines in mercury concentration in a freshwater fishery: isolating the effects of de-acidification and decreased atmospheric mercury deposition in Little Rock Lake. *Science of the Total Environment* **297**, 229-237 (2002). https://doi.org:10.1016/s0048-9697(02)00138-9
18 Zhang, J. *et al.* Mercury in wetlands over 60 years: Research progress and emerging trends. *Science of the Total Environment* **869**, 161862 (2023). https://doi.org:10.1016/j.scitotenv.2023.161862
19 Kelly, C. A., Rudd, J. W. M. & Holoka, M. H. Effect of pH on mercury uptake by an aquatic bacterium: Implications for Hg cycling. *Environmental Science & Technology* **37**, 2941-2946 (2003). https://doi.org:10.1021/es026366o
20 Xu, Z. *et al.* Meteorological drivers of atmospheric mercury seasonality in the temperate northern hemisphere. *Geophysical Research Letters* **49** (2022). https://doi.org:10.1029/2022gl100120
21 Wang, Y. J., Wu, P. P. & Zhang, Y. X. Climate-driven changes of global marine mercury cycles in 2100. *Proceedings of the National Academy of Sciences of the United States of America* **120**, e2202488120 (2023). https://doi.org:10.1073/pnas.2202488120
22 Li, S. Y. *et al.* Dearomatization drives complexity generation in freshwater organic matter. *Nature* **628**, 776–781 (2024). https://doi.org:10.1038/s41586-024-07210-9
23 Balmonte, J. P. *et al.* Sharp contrasts between freshwater and marine microbial enzymatic capabilities, community composition, and DOM pools in a NE Greenland fjord. *Limnology & Oceanography* **65**, 77-95 (2020). https://doi.org:10.1002/lno.11253
24 Wang, X. *et al.* Global warming accelerates uptake of atmospheric mercury in regions experiencing glacier retreat. *Proceedings of the National Academy of Sciences of the United States of America* **117**, 2049-2055 (2020). https://doi.org:10.1073/pnas.1906930117
25 Ahonen, S. A., Hayden, B., Leppänen, J. J. & Kahilainen, K. K. Climate and productivity affect total mercury concentration and bioaccumulation rate of fish along a spatial gradient of subarctic lakes. *Science of the Total Environment* **637**, 1586-1596 (2018). https://doi.org:10.1016/j.scitotenv.2018.04.436
26 Parks, J. M. *et al.* The Genetic Basis for Bacterial Mercury Methylation. *Science* **339**, 1332-1335 (2013). https://doi.org:10.1126/science.1230667

27 Gilmour, C. C. *et al.* Mercury methylation by novel microorganisms from new environments. *Environmental Science & Technology* **47**, 11810-11820 (2013).
28 Liu, Y. R., Delgado-Baquerizo, M., Bi, L., Zhu, J. & He, J. Z. Consistent responses of soil microbial taxonomic and functional attributes to mercury pollution across China. *Microbiome* **6** (2018). https://doi.org:10.1186/s40168-018-0572-7
29 Liu, Y. R. *et al.* Unraveling Microbial Communities Associated with Methylmercury Production in Paddy Soils. *Environmental Science & Technology* **52**, 13110-13118 (2018). https://doi.org:10.1021/acs.est.8b03052
30 Acharya, K. *et al.* Metagenomic water quality monitoring with a portable laboratory. *Water Research* **184** (2020). https://doi.org:10.1016/j.watres.2020.116112
31 Christensen, G. A. *et al.* Development and validation of broad-range qualitative and clade-specific quantitative molecular probes for assessing mercury methylation in the environment. *Applied and Environmental Microbiology* **82**, 6068-6078 (2016). https://doi.org:10.1128/aem.01271-16
32 Ma, M., Du, H. X., Wang, D. Y. & Sun, T. Mercury methylation in the soils and sediments of Three Gorges Reservoir Region. *Journal of Soils and Sediments* **18**, 1100-1109 (2018). https://doi.org:10.1007/s11368-017-1827-9
33 Luton, P. E., Wayne, J. M., Sharp, R. J. & Riley, P. W. The *mcrA* gene as an alternative to 16S rRNA in the phylogenetic analysis of methanogen populations in landfill. *Microbiology-Sgm* **148**, 3521-3530 (2002). https://doi.org:10.1099/00221287-148-11-3521
34 Zhu, C., Zhang, J., Tang, Y., Zhengkai, X. & Song, R. Diversity of methanogenic archaea in a biogas reactor fed with swine feces as the mono-substrate by mcrA analysis. *Microbiological Research* **166**, 27-35 (2011). https://doi.org:10.1016/j.micres.2010.01.004
35 Chen, S., Zhou, Y., Chen, Y. & Gu, J. Fastp: An ultra-fast all-in-one FASTQ preprocessor. *Bioinformatics* **34**, 884-890 (2018). https://doi.org:10.1093/bioinformatics/bty560
36 Magoč, T. & Salzberg, S. L. FLASH: Fast length adjustment of short reads to improve genome assemblies. *Bioinformatics* **27**, 2957-2963 (2011). https://doi.org:10.1093/bioinformatics/btr507
37 Edgar, R. C. UPARSE: Highly accurate OTU sequences from microbial amplicon reads. *Nature Methods* **10**, 996-998 (2013). https://doi.org:10.1038/nmeth.2604
38 Stackebrandt, E. & Goebel, B. M. Taxonomic note: A place for DNA-DNA reassociation and 16S rRNA sequence analysis in the present species definition in bacteriology. *International Journal of Systematic and Evolutionary Microbiology* **44**, 846-849 (1994). https://doi.org:10.1099/00207713-44-4-846
39 Wang, Q., Garrity, G. M., Tiedje, J. M. & Cole, J. R. Naive Bayesian classifier for rapid assignment of rRNA sequences into the new bacterial taxonomy. *Applied and Environmental Microbiology* **73**, 5261-5267 (2007). https://doi.org:10.1128/aem.00062-07
40 IPCC. Climate Change 2023: Sixth Assessment Report (AR6). Intergovernmental Panel on Climate Change. https://www.ipcc.ch/report/ar6/syr/ (2023).
41 Wang, S. *et al.* Comparison of mercury speciation and distribution in the water column and sediments between the algal type zone and the macrophytic type zone in a hypereutrophic lake (Dianchi Lake) in Southwestern China. *Science of the Total Environment* **417**, 204-

213 (2012).
42 Fang, T. *et al.* Distribution, bioaccumulation and trophic transfer of trace metals in the food web of Chaohu Lake, Anhui, China. *Chemosphere* **218**, 1122-1130 (2019). https://doi.org:10.1016/j.chemosphere.2018.10.107
43 Wen, Z. D. *et al.* A national-scale data set for dissolved carbon and its spatial pattern in lakes and reservoirs across China. *Scientific Data* **7** (2020). https://doi.org:10.1038/s41597-020-0419-5
44 Huang, Y., Cui, G. L., Li, B. P., Zhu, X. X. & Yang, Z. Elevated atmospheric CO_2 enhances grazer-induced morphological defense in the freshwater green alga *Scenedesmus obliquus*. *Limnology and Oceanography* **63**, 1004-1014 (2018). https://doi.org:10.1002/lno.10715
45 Blum, P. W., Hershey, A. E., Tsui, M. T. K., Hammerschmidt, C. R. & Agather, A. M. Methylmercury and methane production potentials in North Carolina Piedmont stream sediments. *Biogeochemistry* **137**, 181-195 (2018). https://doi.org:10.1007/s10533-017-0408-8
46 Hintelmann, H., Keppel-Jones, K. & Evans, R. D. Constants of mercury methylation and demethylation rates in sediments and comparison of tracer and ambient mercury availability. *Environmental Toxicology and Chemistry* **19**, 2204-2211 (2000). https://doi.org:10.1002/etc.5620190909
47 Wu, J. *et al.* Water and Sediment Quality in Lakes along the Middle and Lower Reaches of the Yangtze River, China. *Water Resources Management* **26**, 3601-3618 (2012). https://doi.org:10.1007/s11269-012-0093-2
48 Liu, D., Du, Y., Yu, S., Luo, J. & Duan, H. Human activities determine quantity and composition of dissolved organic matter in lakes along the Yangtze River. *Water Research* **168** (2020). https://doi.org:10.1016/j.watres.2019.115132
49 Wu, S. K., Xie, P., Liang, G. D., Wang, S. B. & Liang, X. M. Relationships between microcystins and environmental parameters in 30 subtropical shallow lakes along the Yangtze River, China. *Freshwater Biology* **51**, 2309-2319 (2006). https://doi.org:10.1111/j.1365-2427.2006.01652.x
50 Cui, L., Wei, L. & Wang, J. Residues of organochlorine pesticides in surface water of a megacity in central China: seasonal-spatial distribution and fate in Wuhan. *Environmental Science and Pollution Research* **24**, 1975-1986 (2017). https://doi.org:10.1007/s11356-016-7956-7
51 Wang, W., Ndungu, A. W., Li, Z. & Wang, J. Microplastics pollution in inland freshwaters of China: A case study in urban surface waters of Wuhan, China. *Science of the Total Environment* **575**, 1369-1374 (2017). https://doi.org:10.1016/j.scitotenv.2016.09.213
52 Bai, C. r. *et al.* Geographic Patterns of Bacterioplankton among Lakes of the Middle and Lower Reaches of the Yangtze River Basin, China. *Applied and Environmental Microbiology* **86** (2020). https://doi.org:10.1128/aem.02423-19
53 Zhou, L.-J. *et al.* Trends in the occurrence and risk assessment of antibiotics in shallow lakes in the lower-middle reaches of the Yangtze River basin, China. *Ecotoxicology and Environmental Safety* **183** (2019). https://doi.org:10.1016/j.ecoenv.2019.109511

54 Wang, S. R., Jin, X. C., Zhao, H. C. & Wu, F. C. Phosphorus fractions and its release in the sediments from the shallow lakes in the middle and lower reaches of Yangtze River area in China. *Colloids and Surfaces a-Physicochemical and Engineering Aspects* **273**, 109-116 (2006). https://doi.org:10.1016/j.colsurfa.2005.08.015

55 Li, S., Guo, W. & Mitchell, B. Evaluation of water quality and management of Hongze Lake and Gaoyou Lake along the Grand Canal in Eastern China. *Environmental Monitoring and Assessment* **176**, 373-384 (2011). https://doi.org:10.1007/s10661-010-1590-5
