# Supplementary material for: Elevated atmospheric CO2 decreases methylmercury production in freshwater lakes
Source: Nat Commun. 2025 Dec 26;17:1037. doi: 10.1038/s41467-025-67788-0 (PMC12848011; doi:10.1038/s41467-025-67788-0)
Supplement: Supplementary file 2 — Description of Additional Supplementary Files [file 41467_2025_67788_MOESM2_ESM.pdf]

Description of Additional Supplementary Files

File Name: Supplementary Data 1
Description: **Data sets of observational DOC and MeHg concentrations in global lakes collected from literature.** The publications spanning the period from 2000 to 2023 that the documented MeHg and DOC levels in lake water were retrieved in Web of Science™ on June 6, 2023. From the search results of 201 scientific papers, 49 articles containing 229 samples were obtained for analyses.
